# Decoding the network of *Trypanosoma brucei* proteins that determines sensitivity to apolipoprotein-L1

**DOI:** 10.1371/journal.ppat.1006855

**Published:** 2018-01-18

**Authors:** Rachel B. Currier, Anneli Cooper, Hollie Burrell-Saward, Annette MacLeod, Sam Alsford

**Affiliations:** 1 London School of Hygiene and Tropical Medicine, London, United Kingdom; 2 Institute of Biodiversity, Animal Health and Comparative Medicine, University of Glasgow, Glasgow, United Kingdom; Hunter College, CUNY, UNITED STATES

## Abstract

In contrast to *Trypanosoma brucei gambiense* and *T*. *b*. *rhodesiense* (the causative agents of human African trypanosomiasis), *T*. *b*. *brucei* is lysed by apolipoprotein-L1 (apoL1)-containing human serum trypanolytic factors (TLF), rendering it non-infectious to humans. While the mechanisms of TLF1 uptake, apoL1 membrane integration, and *T*. *b*. *gambiense* and *T*. *b*. *rhodesiense* apoL1-resistance have been extensively characterised, our understanding of the range of factors that drive apoL1 action in *T*. *b*. *brucei* is limited. Selecting our bloodstream-form *T*. *b*. *brucei* RNAi library with recombinant apoL1 identified an array of factors that supports the trypanocidal action of apoL1, including six putative ubiquitin modifiers and several proteins putatively involved in membrane trafficking; we also identified the known apoL1 sensitivity determinants, TbKIFC1 and the V-ATPase. Most prominent amongst the novel apoL1 sensitivity determinants was a putative ubiquitin ligase. Intriguingly, while loss of this ubiquitin ligase reduces parasite sensitivity to apoL1, its loss enhances parasite sensitivity to TLF1-dominated normal human serum, indicating that free and TLF1-bound apoL1 have contrasting modes-of-action. Indeed, loss of the known human serum sensitivity determinants, p67 (lysosomal associated membrane protein) and the cathepsin-L regulator, ‘inhibitor of cysteine peptidase’, had no effect on sensitivity to free apoL1. Our findings highlight a complex network of proteins that influences apoL1 action, with implications for our understanding of the anti-trypanosomal action of human serum.

## Author summary

Expression of the trypanolytic serum component, apolipoprotein-L1 (apoL1), by humans and the concomitant evolution of countermeasures by two African trypanosome sub-species defines their ability to cause disease in humans. A genome-scale RNAi screen identified more than 60 proteins that sensitise non-human infectious trypanosomes to apoL1. Comparing these outputs to previous screens that used different selection approaches provides insights into the mode-of-action of human serum-mediated killing of trypanosomes. The effectiveness of apoL1, and the trypanolytic factors (TLF) that carry it, is dependent on the parasite’s endocytic system for uptake, intracellular transit and delivery to target membranes. Comparing our current data to the outputs from previous screens revealed that apoL1 is able to exploit different routes to access its target membranes, depending on whether it is free or, as in human serum, complexed with TLF1 or TLF2.

## Introduction

*Trypanosoma brucei* ssp. and the related kinetoplastid parasites, *T*. *congolense* and *T*. *vivax*, are endemic to sub-Saharan Africa. *T*. *b*. *gambiense* and *T*. *b*. *rhodesiense* cause an estimated 20,000 cases of human African trypanosomiasis (HAT) per year [1], but as recently as the 1990s there were thought to be up to 500,000 cases per year [2]. While the number of reported cases has dropped significantly in the last decade, approximately 70 million people still live in parts of sub-Saharan Africa regarded as risk areas for HAT [3]. *T*. *b*. *brucei*, *T*. *congolense* and *T*. *vivax* are the causative agents of the cattle wasting disease, nagana, and continue to have a significant impact on agricultural development in the region [4]. While treatments for HAT and animal African trypanosomiasis are available, these exhibit significant toxic side effects, and resistance is becoming an increasing problem in the field [5, 6], making the development of novel therapeutic approaches and a detailed understanding of the mode-of-action of current trypanocidals essential [7].

Most African trypanosomes, including *T*. *congolense*, *T*. *vivax* and *T*. *b*. *brucei*, are sensitive to lysis by human serum. However, the human-infective sub-species, *T*. *b*. *gambiense* and *T*. *b*. *rhodesiense*, have evolved distinct means of evading this innate immunity. Human serum contains two trypanolytic complexes, TLF1 and TLF2 [8–11], both of which include the lytic component, apolipoprotein-L1 (apoL1). ApoL1 forms pores in the trypanosome endosomal and other membranes, a process dependent on conformational change driven by decreasing pH through the parasite’s endosomal-lysosomal system [12–16]. Recent findings suggest that pore opening and subsequent lysis is dependent on return to a neutral pH environment [17]. *T*. *b*. *rhodesiense* is able to evade this lytic attack by expressing the serum resistance-associated (SRA) protein, which binds apoL1 in the endosomal-lysosomal system and prevents pore formation [18, 19]. In contrast, *T*. *b*. *gambiense* resistance to apoL1 is a multifactorial process involving reduced TLF1 uptake by a variant form of the parasite’s haptoglobin-haemoglobin receptor (TbHpHbR) [20–22], *T*. *b*. *gambiense*-specific glycoprotein (TgsGP) mediated endosomal membrane stiffening, and possibly increased lysosomal cysteine protease activity [23, 24]. SRA and TgsGP are related to the parasite’s variant surface glycoprotein (VSG), which is responsible for antigenic variation and immune evasion [25]. However, while the SRA sequence is associated with a specific sub-telomeric *VSG* expression site and its expression varies between *T*. *b*. *rhodesiense* isolates [19], expression of TgsGP by group 1 *T*. *b*. *gambiense* is constitutive, rendering all isolates resistant to lysis by human serum [24].

The efficient uptake of TLF1 by African trypanosomes is dependent on the presence of haptoglobin-related protein in the complex and mediated by the parasite’s HpHb receptor [26–30]. Our understanding of how TLF2 enters the parasite is incomplete. In common with TLF1, it contains haptoglobin-related protein, and can enter *via* the parasite’s HpHb receptor, though much less efficiently than TLF1 [26]. Instead, efficient TLF2 uptake has been proposed to depend upon an interaction between its component IgM and the trypanosome’s VSG surface coat [31]. Until recently, we knew little about the intracellular factors that determine the sensitivity of *T*. *b*. *brucei* to lysis by human serum. Previously, we sought to address this deficit by selecting our bloodstream-form (BSF) *T*. *b*. *brucei* RNAi library in normal human serum (NHS), identifying four key sensitivity determinants [32]. Unsurprisingly, the most significant ‘hit’ was TbHpHbR, depleted by more than 70% of the RNAi target fragments in the selected library. We also identified several other factors: ‘inhibitor of cysteine peptidase’ (ICP) [33], the lysosomal-associated membrane protein, p67 [34], and a putative transmembrane protein of unknown function, Tb927.8.5240. We subsequently showed that ICP modulates the function of TbCATL, a lysosomal cathepsin-L-like cysteine peptidase, and that increased TbCATL activity in *icp* null *T*. *b*. *brucei* renders them less sensitive to trypanolysis by human serum [32]. We speculated that this phenotype was due to increased degradation of TLF or apoL1 by the deregulated lysosomal peptidase [35].

An independent study describing RNAi library screens using NHS and hyperhaptoglobinaemic serum as the selective agents was published in the same year [36]. Using freshly isolated NHS at high concentration the authors identified TbHpHbR, though not p67 or ICP. The addition of high concentration haptoglobin limited the contribution of TbHpHbR to uptake, so reducing the contribution of TLF1 but enhancing that of TLF2 to parasite lysis, and potentially enabling the screen to identify TLF2-specific sensitivity determinants. While a TLF2-specific transporter was not identified in this screen, the role of the parasite’s vacuolar (V-)ATPase and the kinesin, TbKIFC1, in promoting *T*. *b*. *brucei* sensitivity to human serum was recognised [36, 37]. The V-ATPase drives progressive acidification of the endosomal-lysosomal network, a process required for the apoL1 conformational change that enables subsequent integration into the lysosomal membrane and pore formation [12–14]. TbKIFC1 acts to couple lysosomal and mitochondrial membrane permeabilisation, leading to TbEndoG release and nuclear DNA laddering [37]. Intriguingly, the NHS and hyperhaptoglobinaemic serum screens identified non-overlapping sets of proteins, indicating that different selection conditions can have a significant impact on the respective factors identified. This suggests that other important sensitivity determinants remained undiscovered, particularly those that specifically influence the action of apoL1. Given this and the dominance of RNAi target fragments depleting TbHpHbR following selection with NHS [32], we selected our genome-scale BSF *T*. *b*. *brucei* RNAi library in recombinant apoL1, which enters the parasite by non-specific fluid phase endocytosis [15], so bypassing TbHpHbR. Finally, we used two forms of recombinant apoL1, human and baboon; both are effective against *T*. *b*. *brucei*, but the latter is lytic to a wider variety of African trypanosomes, including the human-infective forms, *T*. *b*. *gambiense* and *T*. *b*. *rhodesiense* [38].

The outputs from apoL1-selection of the *T*. *b*. *brucei* RNAi library were broadly similar, irrespective of the primate apoL1 used, suggesting that the mechanism of trypanolytic killing, once either apoL1 has entered the parasite, is indistinguishable. However, comparison with the previous outputs following RNAi library selection in human serum (either normal or hyperhaptoglobinaemic) revealed intriguing similarities and differences. We identified parasite factors that influence *T*. *b*. *brucei* sensitivity to free apoL1 that had previously been implicated in promoting sensitivity to hyperhaptoglobinaemic serum, such as the V-ATPase and TbKIFC1 [36, 37]. However, this comparison also identified NHS-specific (ICP [33] and p67 [34]) and apoL1-specific sensitivity factors. The latter included a group of ubiquitin modifiers, and several proteins with putative roles in membrane trafficking. We propose that, in contrast to TLF1-embedded apoL1, the trypanocidal activity of free apoL1 is not dependent on lysosomal morphology or proteolytic function, as defined by p67 and ICP/TbCATL, respectively. Finally, we present evidence that ubiquitination has a role to play in the regulation of the network of *T*. *b*. *brucei* proteins that support the trypanolytic action of apoL1.

## Results

### A *T*. *b*. *brucei* genome-scale RNAi library identifies apoL1-sensitivity determinants

**Fig 1:**
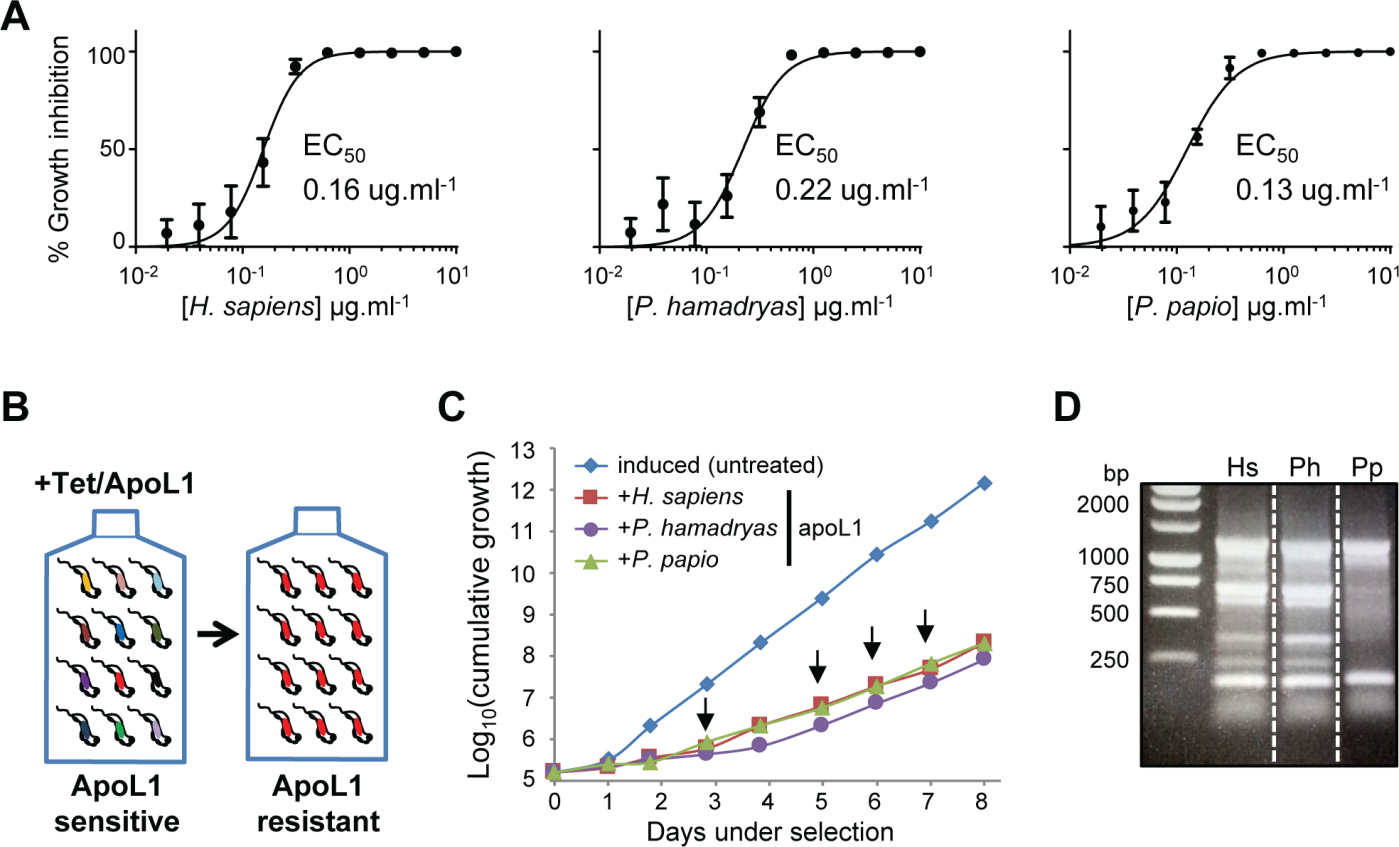
An RNAi library screen to identify *T*. *b*. *brucei* apoL1 sensitivity determinants. (A) *T*. *b*. *brucei* (MITat1.2; strain 2T1) is similarly sensitive to human (*H*. *sapiens*) and baboon (*P*. *hamadryas* and *P*. *papio*) apoL1. EC_50_ assays were carried out in quadruplicate; error bars, standard deviation. (B) Schematic showing selection of apoL1-resistant parasites from the RNAi library. (C) Selection of populations with reduced sensitivity to apoL1. RNAi induced in 1 μg.ml^-1^ tetracycline (Tet) for 24 hours prior to selection (initiated at day-0); arrows indicate culture dilution, and addition of fresh apoL1 and tetracycline at 2X EC_50_ and 1 μg.ml^-1^, respectively. (D) RNAi target fragment-specific PCR amplification from *T*. *b*. *brucei* genomic DNA extracted after eight days’ selection in apoL1 (Hs, *H*. *sapiens*; Ph, *P*. *hamadryas*; Pp, *P*. *papio*).

Selection of BSF *T*. *b*. *brucei* RNAi libraries has previously been used to identify parasite factors that drive the ability of human serum to lyse non-human infective African trypanosomes [32, 36]. These screens identified several endosomal-lysosomal proteins that support the action of TLF; however, neither screen was able to offer direct insight into the proteins that specifically influence the action of apoL1, the lytic component of primate serum TLF. In order to access this level of detail, we selected our genome-scale BSF *T*. *b*. *brucei* RNAi library in recombinant human and baboon (*Papio hamadryas* and *P*. *papio*) apoL1. EC_50_ assays confirmed our strain of *T*. *b*. *brucei* is similarly sensitive to human, *P*. *hamadryas* and *P*. *papio* recombinant apoL1 at 0.16 μg.ml^-1^, 0.22 μg.ml^-1^ and 0.13 μg.ml^-1^, respectively (Fig 1A). In order to enable identification of factors whose loss renders *T*. *b*. *brucei* less sensitive to primate apoL1, expression of the RNAi library was induced for 24 hours prior to the addition of apoL1 at 2X EC_50_, and selection and induction maintained thereafter (Fig 1B). After approximately four days under selection, populations with reduced sensitivity to each recombinant apoL1 emerged, and these maintained slow but consistent growth under continued selection (Fig 1C). Genomic DNA was isolated from these populations on day eight, and subjected to RNAi construct-specific PCR, generating indistinguishable banding patterns for the human and *P*. *hamadryas* apoL1-selected RNAi libraries, and a dissimilar pattern following selection in *P*. *papio* apoL1 (Fig 1D). We sequenced the amplified RNAi target fragment populations from the human and *P*. *papio* apoL1 selected RNAi libraries on an Illumina MiSeq platform (sequencing data is available from the European Nucleotide Archive, www.ebi.ac.uk/ena, accession number PRJEB23779).

**Fig 2:**
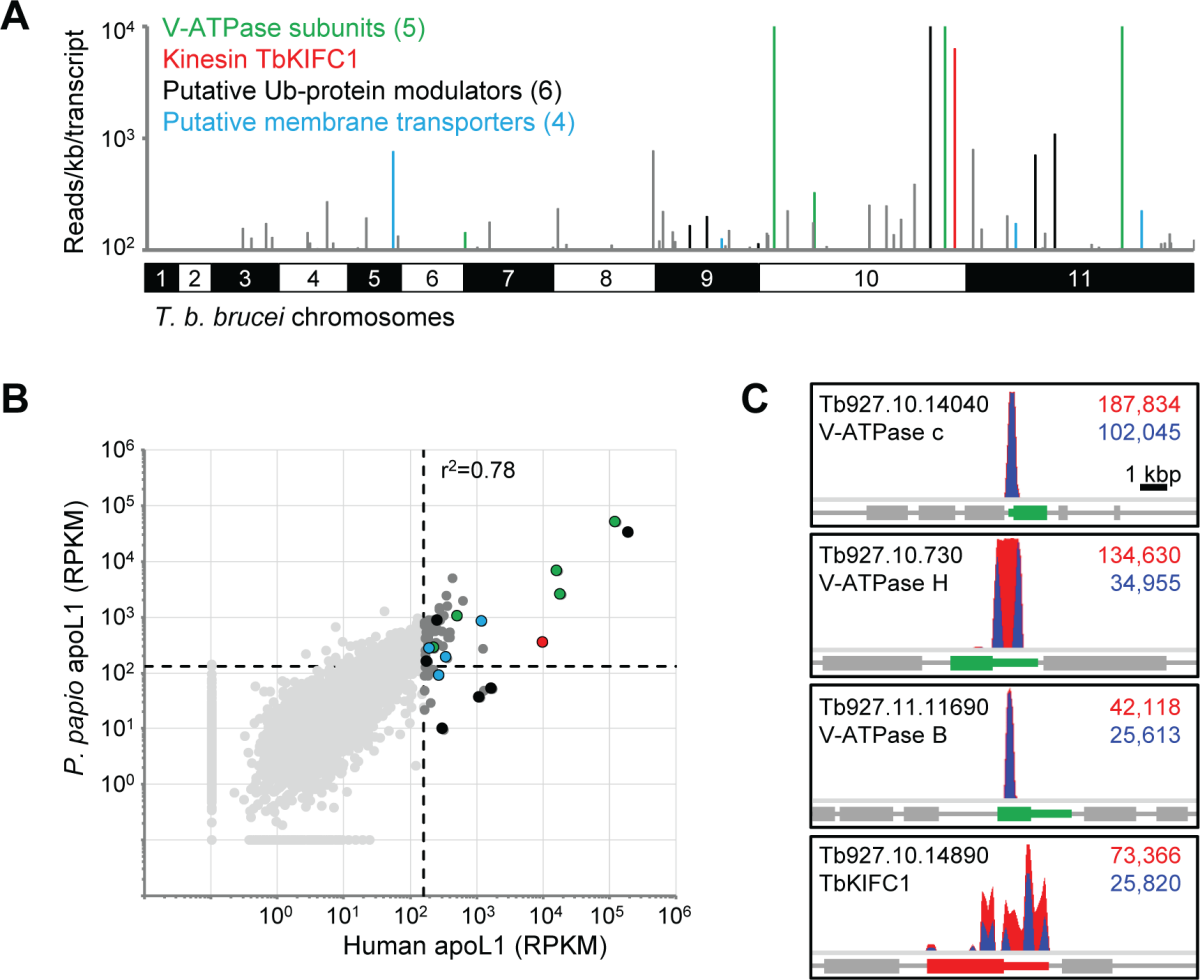
Selecting a genome-scale *T*. *b*. *brucei* RNAi library identifies parasite determinants of apoL1 sensitivity. (A) Genome-wide human apoL1 RIT-seq profile representing 7,398 non-redundant predicted transcripts, showing those targeted by 100 or more reads per kilobase containing the 14-base RNAi construct barcode sequence. ‘Hits’ targeting V-ATPase subunits and the kinesin, TbKIFC1, are highlighted in green and red, respectively; novel high confidence hits corresponding to six putative ubiquitin modifiers and four putative membrane transporters are highlighted in black and blue, respectively (see Fig 4 and S1 Table for further details). (B) RNAi library selection with human or *P*. *papio* apoL1 identified similar sets of sensitivity determinants (r^2^ = 0.78); key hits are coloured as in (A), and are similarly abundant in both selected libraries. Data presented as RPKM (plus 0.1; reads per kilobase of transcript per million mapped reads) to correct for variations in read depth between the respective RNAi library screens (and to enable plotting of zero read outputs on log_10_ scales). Dashed lines represent the 100-read stringency criterion converted to RPKM for each RNAi library screen (human apoL1, 158; *P*. *papio* apoL1, 132). (C) RIT-seq profiles for three of the V-ATPase subunits and for TbKIFC1; predicted transcripts (open reading frames and untranslated regions) are coloured as in (A); total reads (red) and tagged reads (blue; containing the 14-base RNAi construct barcode sequence) mapped to each predicted transcript are shown in the top right corner of each panel.

Using our established RIT-seq methodology [39], we generated and mapped individual sequence reads representing the human apoL1-enriched RNAi target fragments, constituting about 1.65 million reads. Approximately 38% of these reads included the 14-base RNAi construct-specific barcode found at the junction of each gene-specific RNAi target fragment. This enabled us to identify the ‘high confidence hits’, i.e. those represented by more than 99 reads per kilobase per predicted transcript (open reading frames plus predicted untranslated regions, as annotated in the TREU927 reference genome available at www.tritrypdb.org) containing the RNAi construct barcode, and at least two independent RNAi target fragments. We have previously applied similarly stringent criteria to define the efficacy determinants of the anti-HAT drugs [40] and NHS [32]. This analysis revealed that selection in human apoL1 had enriched for a complex set of RNAi target fragments (Fig 2A and S1 Table). The outputs following *T*. *b*. *brucei* RNAi library selection in *P*. *papio* apoL1 constituted 1.68 million reads, of which approximately 45% included the 14-base RNAi construct-specific barcode.

To enable comparison of the outputs following selection in human and *P*. *papio* apoL1, we converted absolute reads to RPKM (reads per kilobase per transcript per million mapped reads); the 100-read stringency criteria were also converted to RPKM for each screen (human apoL1, 158; *P*. *papio* apoL1, 132; dashed lines in Fig 2B). Although there were differences in the patterns obtained following RNAi construct-specific PCR (Fig 1D), high throughput sequencing revealed that growth in either human or *P*. *papio* apoL1 selected for highly similar sets of RNAi fragments (r^2^ = 0.78; Fig 2B). This data, in conjunction with the shared RNAi construct-specific PCR pattern following RNAi library selection in human or *P*. *hamadryas* apoL1 (Fig 1D), suggests that apoL1 from the three species is dependent on a similar array of *T*. *b*. *brucei* sensitivity determinants. Therefore, our subsequent analyses focused on the outputs obtained following human apoL1-selection of the *T*. *b*. *brucei* RNAi library. These constituted 63 ‘hits’ represented by more than 99 reads per kilobase per transcript, of which 39 aligned with more than one RNAi target fragment (S1 Table).

### ApoL1 selection identifies known TLF2 sensitivity determinants

The top five ‘hits’ identified by apoL1 selection included the kinesin, TbKIFC1, and three subunits of the V-ATPase complex (two additional subunits also feature in our ‘hit’ list; Fig 2 and S1 Table). A previous RNAi library screen using hyperhaptoglobinaemic human serum as the selective agent, to enrich for TLF2-interacting proteins, also identified TbKIFC1 and three V-ATPase subunits (V_1_-F and -H, and V_0_-a) [36, 37]. Interestingly, though our screen identified five V-ATPase subunits, V_0_-c and V_1_-B, -C, -D and–H, the latter was the only subunit common to both screens. Two of the five V-ATPase subunits (Tb927.10.14040 and Tb927.11.11690) identified following apoL1 selection failed to fulfil our stringency criteria for assignment as high-confidence apoL1 sensitivity determinants, being represented by only one RNAi target fragment each (Fig 2C; S1 Table). However, identification of RNAi target fragments for multiple V-ATPase subunits in the selected library, indicate that these likely constitute genuine apoL1 sensitivity determinants.

A review of our previously published genome-scale phenotyping analysis [41], revealed that depletion of Tb927.10.14040 (V_0_-c) and Tb927.11.11690 (V_1_-B) led to a significant loss of fitness in BSF *T*. *b*. *brucei* after three days (S1 Table). Therefore, these proteins are likely essential for parasite survival, contributing to the limited number of independent RNAi target fragments mapping to the cognate genes following selection in apoL1. To confirm the importance of Tb927.10.14040 for *T*. *b*. *brucei* growth and apoL1 sensitivity, we generated three independent 2T1 *T*. *b*. *brucei* cell lines expressing stemloop RNAi cassettes targeting this transcript; the 2T1 strain of BSF *T*. *b*. *brucei* has an integration landing pad on chromosome 2, enabling consistent inducible expression between clones from integrated cassettes [42, 43]. As predicted, Tb927.10.14040 RNAi knockdown led to a significant growth defect, which we were able to modulate by reducing the amount of tetracycline used to induce stemloop RNAi cassette expression (S1A Fig). Depletion of Tb927.10.14040 following induction in 2 ng.ml^-1^ tetracycline resulted in increased survival in the presence of 10 μg.ml^-1^ apoL1 and 0.1% NHS, confirming the contribution of this putative V-ATPase subunit to parasite apoL1 sensitivity (S1B Fig) and highlighting its general role in determining sensitivity to human serum (S1C Fig). This data further emphasises the importance of the V-ATPase complex to apoL1-mediated killing of *T*. *b*. *brucei* [36].

### TbCATL and p67 do not influence *T*. *b*. *brucei* sensitivity to apoL1

The identification of predicted apoL1-sensitivity determinants (TbKIFC1 and subunits of the V-ATPase) provides a robust validation of our RNAi library screening approach. However, our screen did not identify two proteins previously shown to contribute to *T*. *b*. *brucei* sensitivity to NHS: ‘inhibitor of cysteine peptidase’ (ICP) and the lysosomal-associated membrane protein, p67 [24, 32, 34]. ICP determines the trypanolytic efficacy of NHS *via* its regulation of the parasite’s cathepsin-L-like cysteine protease, TbCATL [32]; this finding led to the hypothesis that lysosomal TbCATL is able to limit human serum efficacy through degradation of apoL1, TLF or both [35]. In contrast, p67, contributes to the maintenance of lysosomal morphology, and depletion of this protein leads to changes in lysosome structure and a concomitant reduction in sensitivity to lysis by human serum [34].

**Fig 3:**
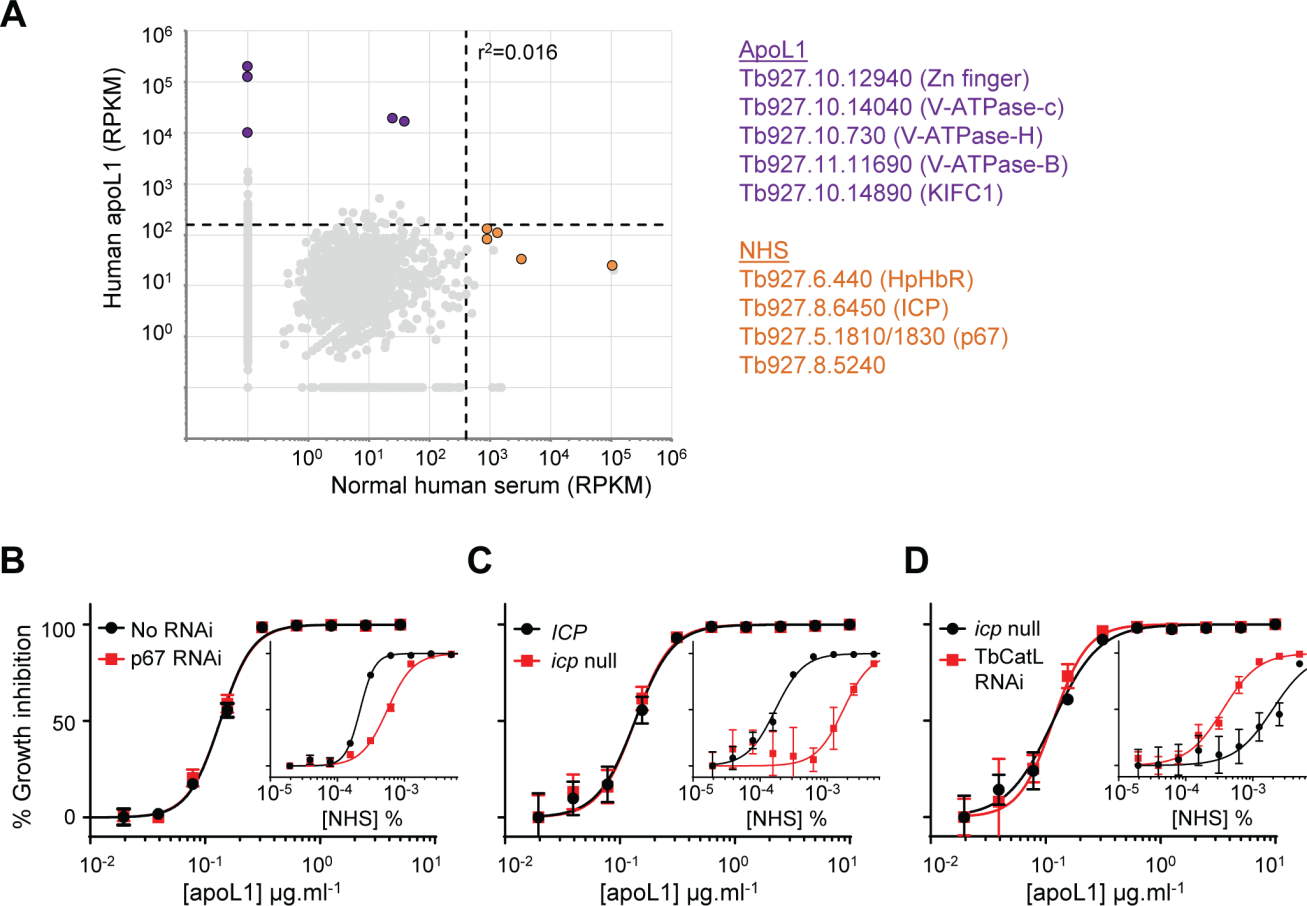
Distinct sets of *T*. *b*. *brucei* proteins determine parasite sensitivity to apoL1 and NHS. (A) Reads containing the 14-base RNAi construct-specific barcode identified following RNAi library selection in human apoL1 (this study) or NHS [32] plotted as RPKM (plus 0.1) to correct for variations in read depth between the respective RNAi library screens (and to enable plotting of zero read outputs on log_10_ scales); r^2^ = 0.016. Dashed lines represent the 100-read stringency criterion converted to RPKM for each RNAi library screen (human apoL1, 158; NHS, 404); apoL1 (purple) and NHS (orange)-specific RNAi targets are highlighted and the top hits listed (gene ID and functional annotation; see S2 and S3 Tables for further details). (B-D) Representative EC_50_ assays carried out in quadruplicate confirming that (B) p67 knockdown, (C) *ICP* deletion and (D) TbCATL depletion does not affect *T*. *b*. *brucei* sensitivity to apoL1. Insets, representative EC_50_ assays carried out in quadruplicate showing the known impact of the corresponding manipulations on sensitivity to NHS. Error bars, standard deviation.

To enable a comparison with the current data set, we reanalysed the outputs from our original NHS RNAi library screen [32] (European Nucleotide Archive, www.ebi.ac.uk/ena, accession number PRJEB23776), generating reads per kilobase per transcript values for each annotated gene. Previously, the outputs were expressed as reads per kilobase per CDS; this reanalysis ensured compatibility with the current data set and recognised that targeted destruction of any part of a transcript will likely lead to depletion of the cognate protein (no additional high-confidence ‘hits’ were identified). To correct for differences in read depth between the two experiments, we converted the NHS and apoL1 data sets to RPKM (Fig 3A); the 100-read stringency criteria were also converted to RPKM for each screen (human apoL1, 158; NHS, 404; dashed lines in Fig 3A). No RNAi target fragment surpassed the corrected ‘100-read’ stringency criterion in both screens, suggesting that distinct sets of proteins determine the sensitivity of *T*. *b*. *brucei* to apoL1 and NHS (Fig 3A; S2 and S3 Tables).

While it is unsurprising that *T*. *b*. *brucei* sensitivity to apoL1 is unaffected by loss of the HpHb receptor (uptake of free apoL1 occurs independently of this receptor *via* non-specific fluid phase endocytosis [15]), we were surprised that RNAi targeting fragments corresponding to ICP and p67 were not more prominent following apoL1 selection. Both p67 and ICP failed to fulfil our stringency criteria for assignment as high-confidence apoL1 sensitivity determinants. Following selection in human (and *P*. *papio*) apoL1, the two predicted p67 transcripts returned RPKM values of 104 (41) and 80 (31), respectively (S2A Fig; S3 Table); while, the predicted *ICP* transcript returned an RPKM value of 32 (32) (S2B Fig; S3 Table). In both cases, the RPKM values were less than the RPKM 99-read stringency cut-off (human apoL1, 158; *P*. *papio* apoL1, 132).

These data strongly suggest that, in contrast to NHS, neither p67 nor ICP has a significant role in determining the sensitivity of *T*. *b*. *brucei* to apoL1. To test this hypothesis, we revisited our published p67 stemloop RNAi and *icp* null cell lines [32, 40]. As predicted, RNAi knockdown of p67 had no effect on parasite sensitivity to apoL1; however, as previously shown [34], depletion of p67 had a significant impact on parasite sensitivity to NHS, reducing it more than two-fold (Fig 3B). Consistent with our previous findings [32], *icp* null *T*. *b*. *brucei* were approximately ten-fold less sensitive to NHS, but loss of *ICP* had no significant impact on parasite sensitivity to apoL1 (Fig 3C). Moreover, while depletion of TbCATL in *icp* null *T*. *b*. *brucei* complemented the loss of NHS sensitivity, as previously shown [32], it had no effect on parasite sensitivity to apoL1 (Fig 3D). These data confirm that free apoL1 is not vulnerable to intracellular TbCATL-mediated proteolytic attack, and indicate that lysosomal function, as defined by p67, ICP and TbCATL, has no significant impact on *T*. *b*. *brucei* killing by free apoL1.

### RNAi library selection identifies novel *T*. *b*. *brucei* apoL1 sensitivity determinants

As well as the known apoL1/TLF2 sensitivity determinants described above, our RNAi library screens also identified a large number of novel factors, which may contribute to apoL1 action (S1 Table). Twenty-four of the identified proteins are annotated ‘hypothetical’, making it difficult to speculate on their mode of interaction with apoL1. Of the remaining proteins with a functional annotation, two groups stood out: putative ubiquitin modifiers and proteins associated with vesicle and membrane trafficking (Fig 2A).

**Fig 4:**
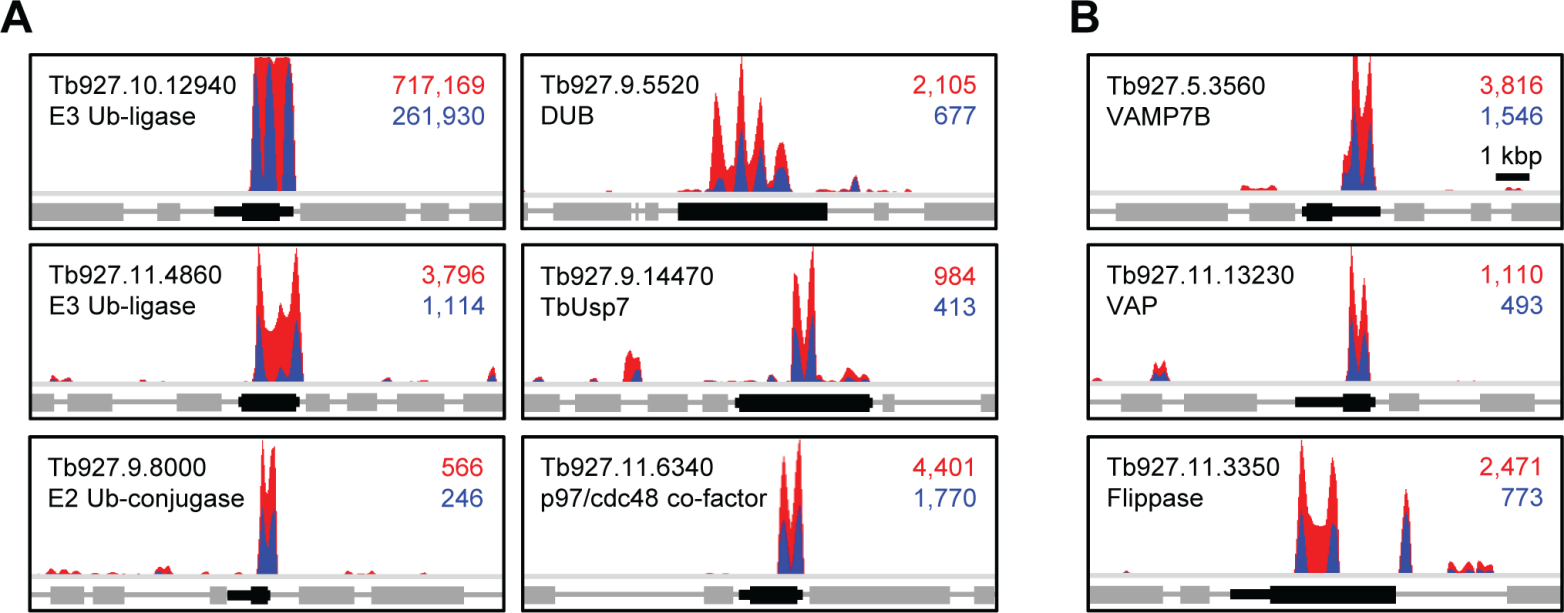
RIT-seq profiles of candidate novel apoL1-sensitivity determinants. ApoL1 sensitivity determinants include (A) putative ubiquitin modifiers and (B) putative membrane trafficking proteins. RIT-seq profiles show predicted transcripts (open reading frames and untranslated regions of interest in black) and the RNAi targeting fragment reads mapped; total reads (red) and tagged reads (blue; containing the 14-base RNAi construct barcode sequence) mapped to each predicted transcript are shown in the top right corner of each panel. Known or putative annotations based on domain organisation are included beneath each accession number and derived from GeneDB (http://www.genedb.org/Homepage/Tbruceibrucei927). Ub, ubiquitin; DUB, deubiquitinase; VAMP, vesicle-associated membrane protein; VAP, VAMP-associated protein.

We identified six putative ubiquitin modifiers in our screen: two Zn-RING finger E3-ubiquitin ligases, an E2-ubiquitin conjugase, two ubiquitin hydrolases (or deubiquitinases, DUBs), and a PUB-domain containing protein (a putative p97/cdc48 cofactor [44]) (Fig 4A; S1 Table). Ubiquitination plays a role in determining the efficacy of the anti-HAT drug, suramin [40, 45]; however, this is the first report of its involvement in apoL1 action against *T*. *b*. *brucei*. The recent finding that apoL1-mediated killing of *T*. *b*. *brucei* involves mitochondrial membrane permeabilisation [37] highlighted the importance of the transit of apoL1-containing membrane from the endosomal-lysosomal system to the mitochondrion. Consistent with this, we identified four uncharacterised proteins with putative roles in membrane trafficking: Tb927.5.3560 (vesicle associated membrane protein, VAMP7B), Tb927.11.13230 (‘major sperm protein’, MSP domain; characteristic of VAMP-associated proteins), Tb927.9.9750 (‘secretory carrier membrane protein’, SCAMP domain) and an aminophospholipid-transporting ATPase (or ‘flippase’; Tb927.11.3350) (Fig 4B; S1 Table).

### A putative lysosomal E3-ubiquitin ligase influences *T*. *b*. *brucei* sensitivity to apoL1 and NHS

**Fig 5:**
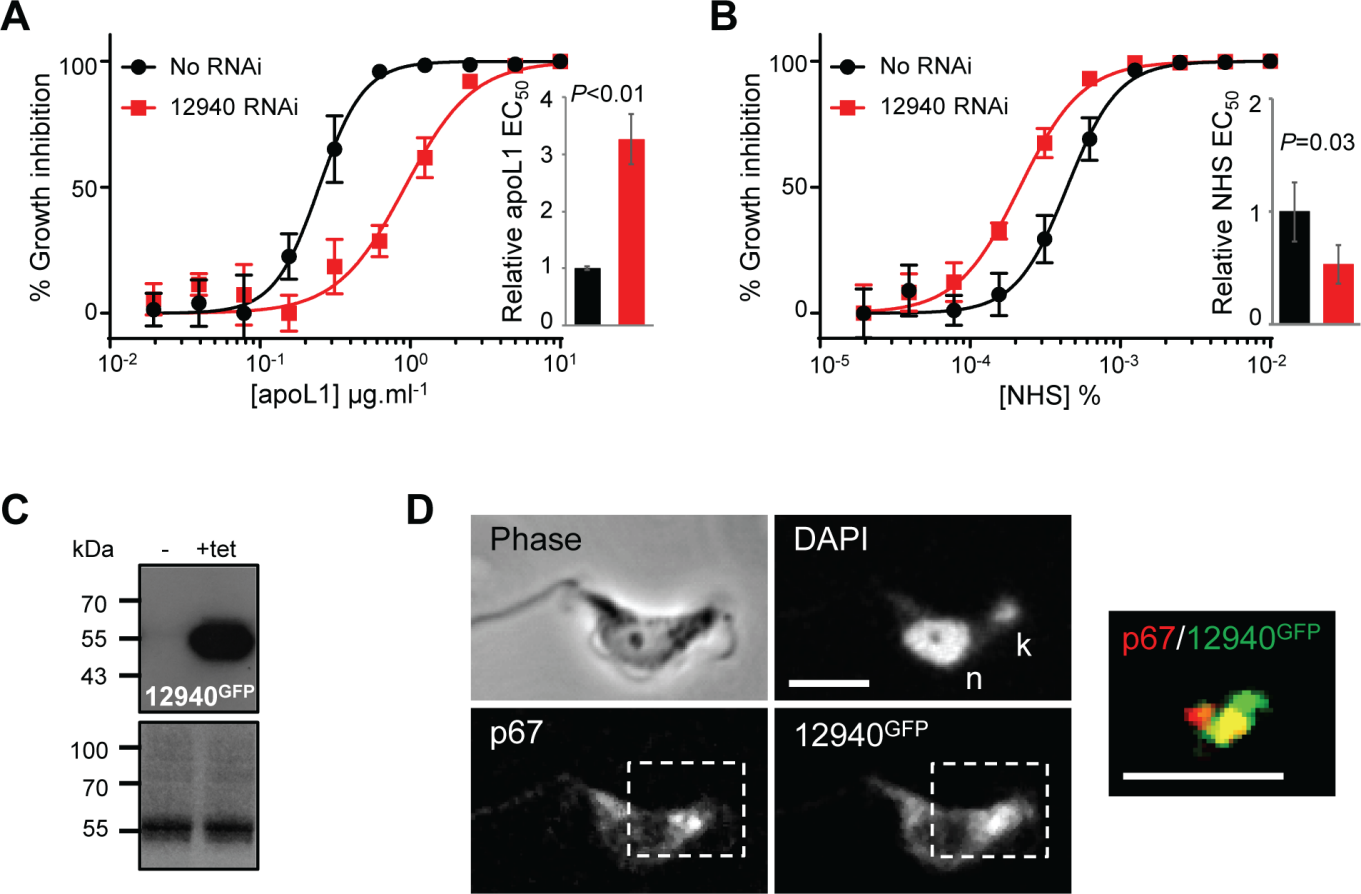
Tb927.10.12940 has opposing effects on apoL1 and NHS sensitivity, and localises to the lysosome. Representative EC_50_ assays carried out in quadruplicate showing the impact of Tb927.10.12940 RNAi knockdown on *T*. *b*. *brucei* sensitivity to (A) apoL1 and (B) NHS. Inset charts show pooled EC_50_ data for three independent cell lines. Error bars, standard deviation; *P*-values derived from paired students t-test. (C) Western blot showing tetracycline (tet)-inducible expression of Tb927.10.12940^GFP^. (D) Immunofluorescence localisation of Tb927.10.12940^GFP^ and the lysosomal membrane protein, p67; counter-staining with the DNA intercalating dye, DAPI, reveals the kinetoplast (k) and nucleus (n). A processed merged image of the region of interest shows the co-localisation of Tb927.10.12940^GFP^ and p67. Scale bar, 5 μm.

The putative Zn RING finger E3-ubiquitin ligase, Tb927.10.12940, was the top ‘hit’ following RNAi library selection in human apoL1 (and second, following selection in *P*. *papio* apoL1); it was targeted by >700,000 reads, of which >261,000 contained the RNAi construct-specific barcode (Fig 4A; S1 Table). Targeted knockdown of Tb927.10.12940 by stemloop RNAi in 2T1 *T*. *b*. *brucei* had no detectable effect on parasite population growth (S3A Fig), but resulted in a more than three-fold increase in apoL1 EC_50_ (Fig 5A), confirming that this putative E3-ubiquitin ligase is an apoL1 sensitivity determinant. Interestingly, knockdown of Tb927.10.12940 rendered BSF *T*. *b*. *brucei* approximately two-fold more sensitive to NHS (Fig 5B), explaining why RNAi library selection in human serum did not identify this protein [32]. Ectopic expression of a *C*-terminally GFP-tagged copy of Tb927.10.12940 (12940^GFP^; Fig 5C) revealed a discrete signal between the kinetoplast and nucleus, reminiscent of an endocytic compartment (S3B Fig), which was confirmed as the lysosome by co-localisation with the lysosomal membrane protein, p67 [34] (Fig 5D).

The validation of Tb927.10.12940 as an apoL1 sensitivity determinant and the presence of other putative ubiquitin modifiers in the ‘hit’ list (six of 63 ‘hits’; Fig 2A; S1 Table), suggests that ubiquitination may contribute to *T*. *b*. *brucei* apoL1 sensitivity. At present, it is unclear which parasite proteins are modified and how this influences apoL1 action. However, using the online UbPred algorithm [46] we identified high confidence candidate ubiquitination sites in 23 of the 63 top hits identified following apoL1 selection of our BSF *T*. *b*. *brucei* RNAi library (S4 Table). Therefore, changes in the ubiquitination status of one or more of these proteins may underlie the changes in apoL1 sensitivity observed following RNAi-mediated knockdown of Tb927.10.12940 (Fig 5A and 5B).

### Evidence for Tb927.10.12940 and Tb927.9.8000 interdependence

**Fig 6:**
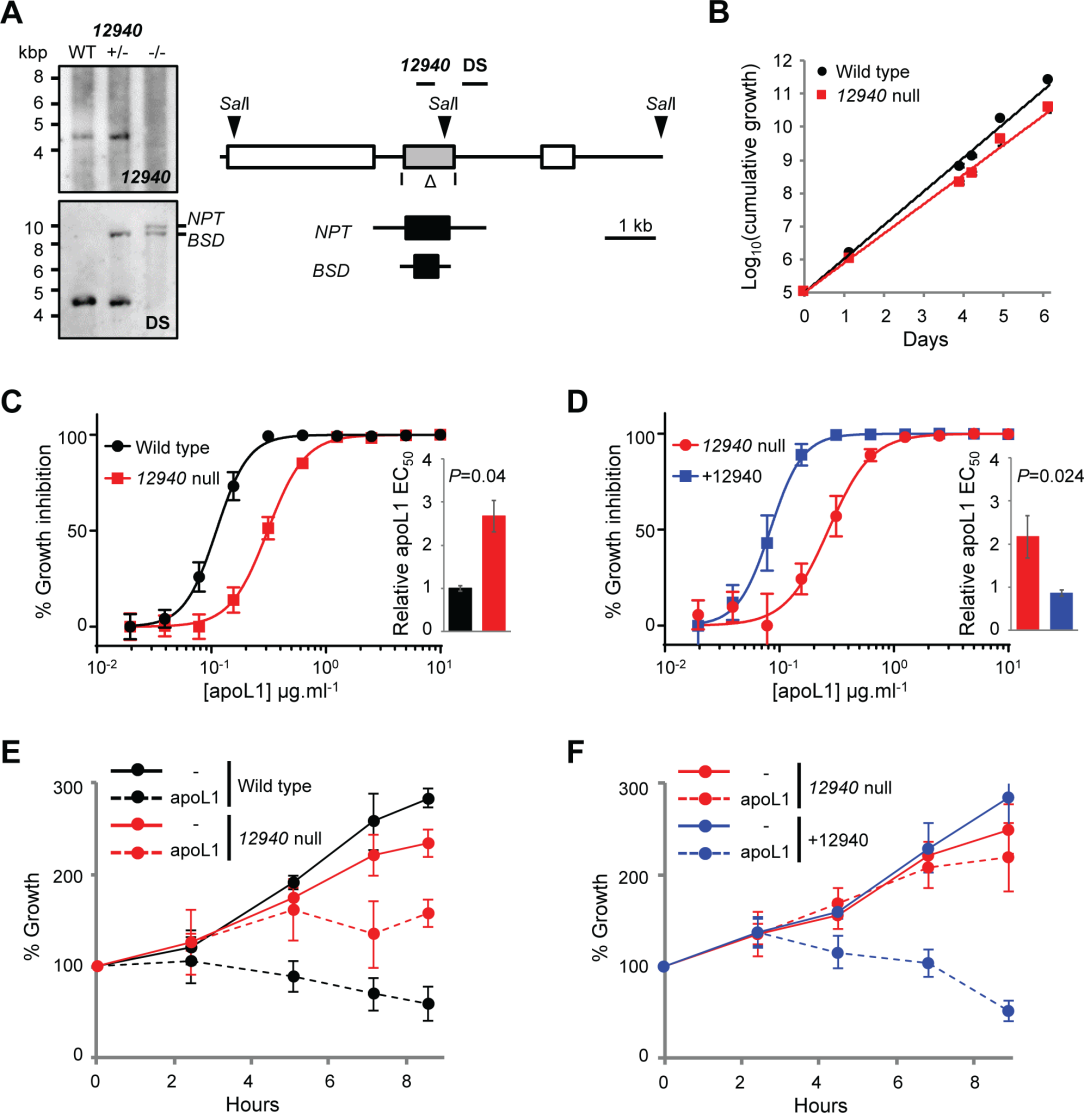
Generation and complementation of *12940* null *T*. *b*. *brucei*. (A) Southern blot confirming *Tb927*.*10*.*12940* deletion; *BSD*, blasticidin-S-deaminase; *NPT*, neomycin phosphotransferase. Schematic showing segment deleted from the *Tb927*.*10*.*12940* locus (open triangle); positions of *Sal*I restriction sites (arrowheads), deletion (12940) and flanking (DS) probes highlighted. (B) Cumulative growth of wild type and three independent *12940* null *T*. *b*. *brucei* cell lines; error bars showing standard deviation are smaller than the plot symbols. (C, D) Representative EC_50_ assays carried out in quadruplicate showing the impact of (C) *Tb927*.*10*.*12940* deletion and (D) re-expression on apoL1 sensitivity. Insets, pooled EC_50_ data for at least two independent cell lines. (E, F) Kinetic analyses of parasite killing in 10 μg.ml^-1^ apoL1 following (E) *Tb927*.*10*.*12940* deletion and (F) re-expression; each assay was carried out using three independent cell lines, re-expression was induced in 1 μg.ml^-1^ tetracycline for 24 hours prior to exposure to apoL1. Error bars, standard deviation; *P*-values derived from paired students t-test.

In order to explore whether Tb927.10.12940 acts in concert with any of the other putative ubiquitin modifiers identified in our screen or affects the action of other apoL1 sensitivity determinants, we generated a *12940* null 2T1 *T*. *b*. *brucei* following integration of neomycin phosphotransferase (*NPT*) and blasticidin-S-deaminase (*BSD*) selectable markers (Fig 6A). In contrast to RNAi knockdown, deletion of both copies of the *Tb927*.*10*.*12940* open reading frame led to an increased population doubling time (wild type, 7.0±0.07 hours; *12940* null, 8.0±0.04 hours; Fig 6B). However, consistent with our earlier data, *12940* null *T*. *b*. *brucei* were less sensitive to apoL1 than wild type parasites (Fig 6C), a phenotype complemented by 12940 re-expression (Fig 6D). Furthermore, deletion of *12940* reduced the speed of parasite killing in the presence of 10 μg.ml^-1^ apoL1 (Fig 6E), a concentration comparable with that found in human serum [15]; this phenotype was complemented by 12940 re-expression (Fig 6F).

E3-ubiquitin ligases act to direct the E2-ubiquitin conjugases to modify specific protein targets [47]. RNAi library selection in apoL1 identified one putative E2-ubiquitin conjugase, Tb927.9.8000, leading us to speculate that the influence of Tb927.10.12940 on *T*. *b*. *brucei* apoL1 and NHS sensitivity would be dependent on this protein. Therefore, these proteins may exhibit an epistatic interaction. To test this hypothesis, we knocked down Tb927.9.8000 by stemloop RNAi in wild type and *12940* null 2T1 *T*. *b*. *brucei*. We also *N*-terminally GFP-tagged Tb927.9.8000 at the native locus, enabling tracking of protein knockdown (S4A Fig), but were unable to detect a specific localisation of the fusion protein. RNAi knockdown of Tb927.9.8000 in either wild type (S4B Fig) or *12940* null 2T1 *T*. *b*. *brucei* (S4C Fig) led to a significant growth defect; deletion of *Tb927*.*10*.*12940* did not enhance the observed growth defect (wild type, Tb927.9.8000 RNAi, 9.3±1.2 hours; *12940* null, Tb927.9.8000 RNAi, 9.7±0.4 hours; S5D Fig).

**Fig 7:**
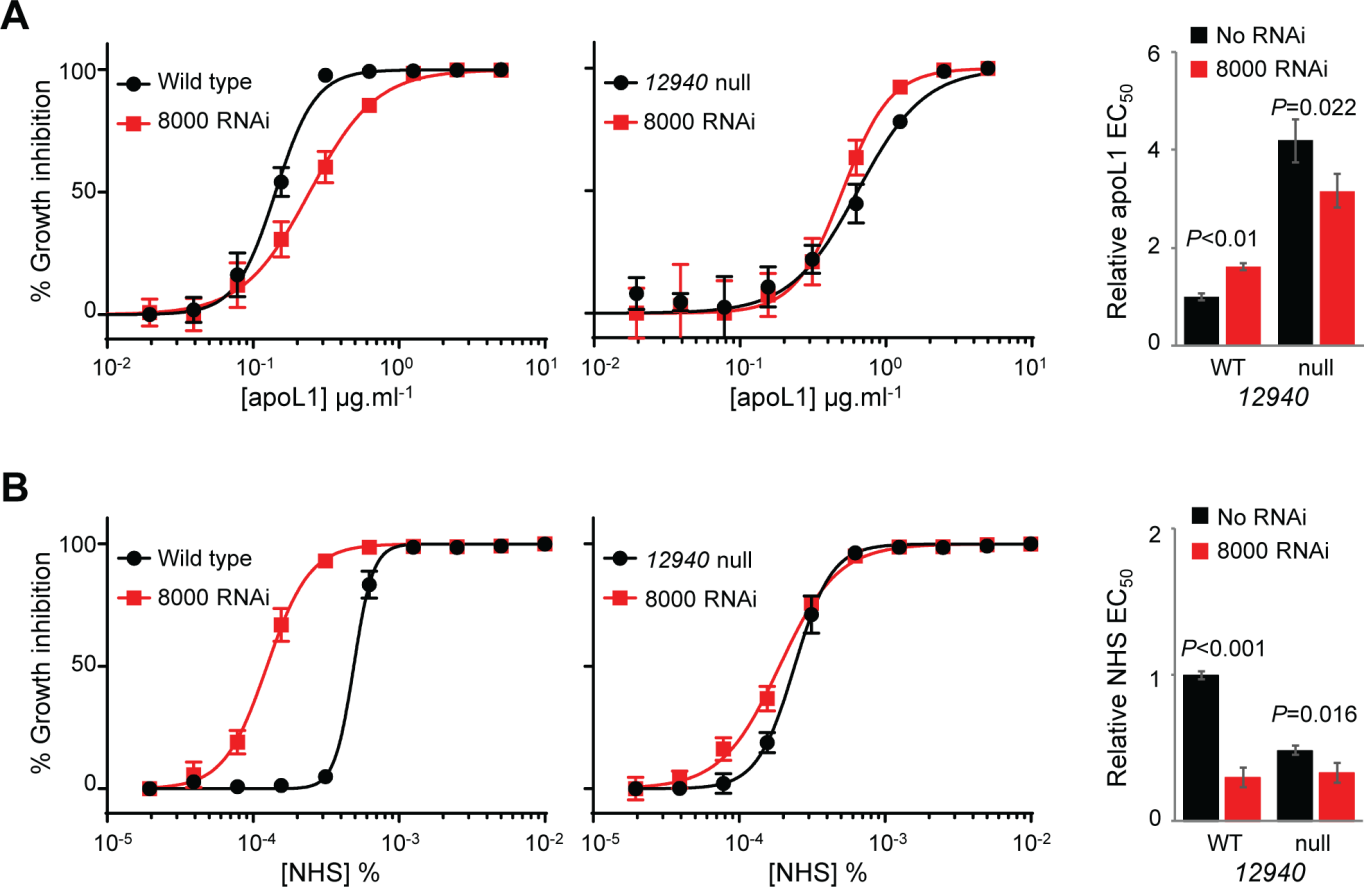
Evidence for interdependence between Tb927.9.8000 and Tb927.10.12940. Representative EC_50_ assays carried out in quadruplicate showing the impact of Tb927.9.8000 RNAi knockdown in wild type (left hand panels) and *12940* null (middle panels) *T*. *b*. *brucei* on (A) apoL1 and (B) NHS sensitivity. Right hand panels, pooled EC_50_ data for at least three independent cell lines. Error bars, standard deviation.

RNAi knockdown of Tb927.9.8000 in wild type 2T1 *T*. *b*. *brucei* led to a slight but significant reduction in apoL1 sensitivity; however, Tb927.9.8000 RNAi did not further reduce the apoL1 sensitivity of *12940* null 2T1 *T*. *b*. *brucei* (Fig 7A). Tb927.9.8000 depletion also affected NHS sensitivity, resulting in a three-fold EC_50_ decrease, though again, there was no enhancement of this effect in *12940* null 2T1 *T*. *b*. *brucei* (Fig 7B). The absence of any significant additive effect, following loss of both proteins, upon growth or apoL1 and NHS sensitivity, suggests that Tb927.10.12940 and Tb927.9.8000 act together to influence the trypanocidal action of these agents.

### Tb927.10.12940 and TbKIFC1 exhibit trypanocide-specific interdependence

While Tb927.10.12940 and Tb927.9.8000 may act together to influence the trypanocidal activity of apoL1 and NHS, they are unlikely to interact directly with TLF or apoL1. Rather, we hypothesised that their putative ubiquitin ligase and conjugase functions enable them to modulate the activity of apoL1/TLF-interacting proteins. Given the predominantly lysosomal localisation of 12940^GFP^ (Fig 5D), other proteins localising to this organelle might be functionally dependent on Tb927.10.12940 and Tb927.9.8000. One such candidate protein is TbKIFC1, which though not limited to the lysosome, directs apoL1-containing membrane from the lysosome to the mitochondrion [37]; this protein is also a putative suramin efficacy determinant [40]. Our analysis using the UbPred algorithm [46] identified four putative high confidence ubiquitination sites in TbKIFC1 (S4 Table), suggesting that ubiquitination, possibly mediated by Tb927.10.12940 and Tb927.9.8000, might play a role in modulating its ability to influence *T*. *b*. *brucei* apoL1, NHS and suramin sensitivity.

**Fig 8:**
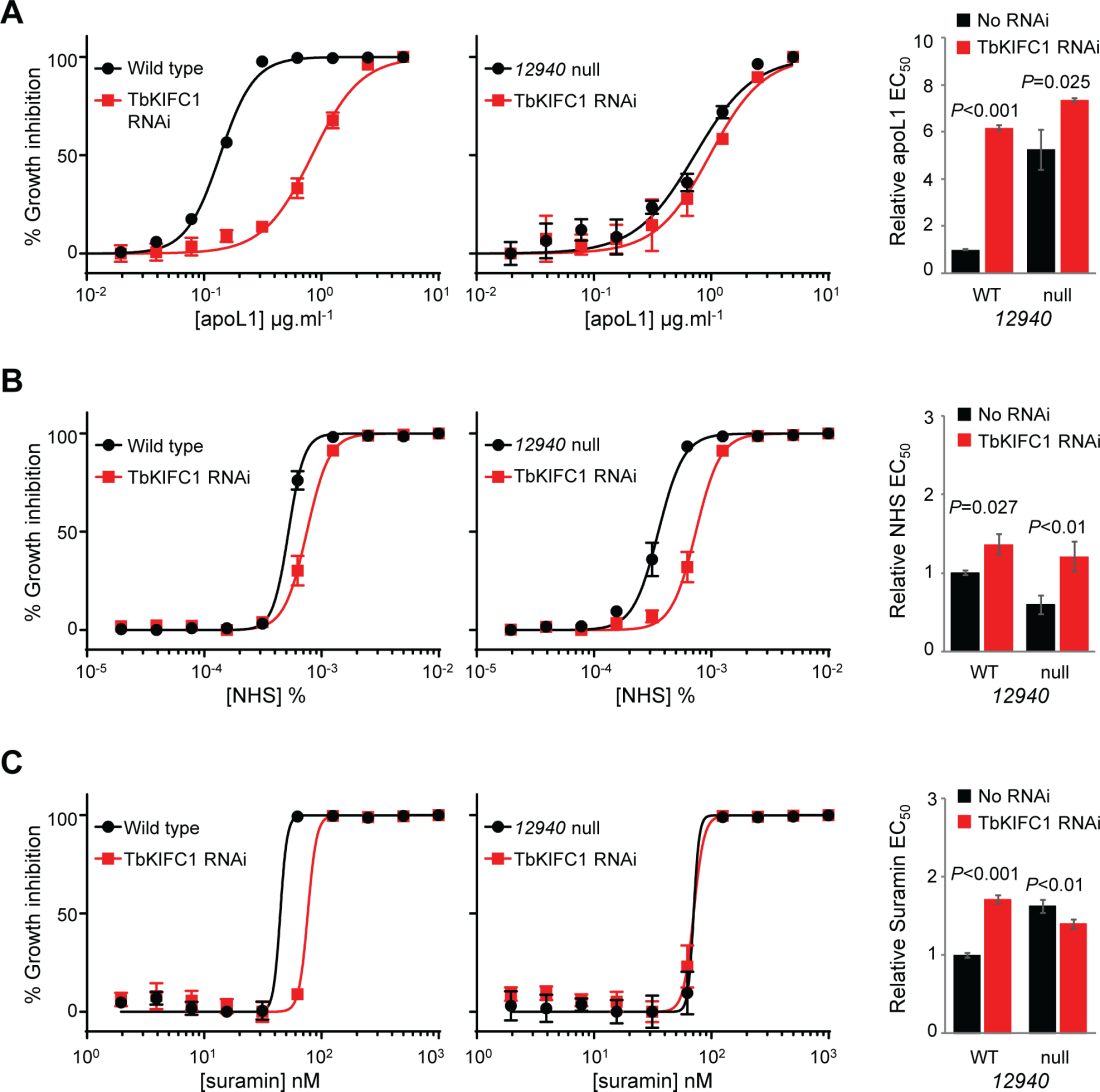
TbKIFC1 depletion complements NHS sensitivity of *12940* null *T*. *b*. *brucei*, but has no additive effect on apoL1 sensitivity or suramin efficacy. Representative EC_50_ assays carried out in quadruplicate showing the impact of TbKIFC1 RNAi knockdown in wild type (left hand panels) and *12940* null (middle panels) *T*. *b*. *brucei* on (A) apoL1, (B) NHS and (C) suramin sensitivity. Right hand panels, pooled EC_50_ data for at least three independent cell lines. Error bars, standard deviation.

RNAi knockdown of TbKIFC1 in *12940* null 2T1 *T*. *b*. *brucei* had no additive impact on population growth (S5 Fig), but had different effects on the trypanocidal activity of free apoL1, NHS and suramin (Fig 8). RNAi knockdown of TbKIFC1 in wild type 2T1 *T*. *b*. *brucei* led to an approximately six-fold increase in apoL1 EC_50_, while concurrent loss of *Tb927*.*10*.*12940* had a marginal additive effect (Fig 8A). In contrast, even though *12940* null 2T1 *T*. *b*. *brucei* are more sensitive to NHS, RNAi knockdown of TbKIFC1 complemented this sensitisation, reducing NHS sensitivity to the same level seen following TbKIFC1 RNAi knockdown in wild type 2T1 *T*. *b*. *brucei* (Fig 8B). TbKIFC1 depletion rendered wild type 2T1 *T*. *b*. *brucei* 1.7-fold less sensitive to suramin, validating its identification following suramin selection of the RNAi library [40]. Unexpectedly, we found that *12940* null 2T1 *T*. *b*. *brucei* were 1.6-fold less sensitive to suramin than wild type parasites. A reanalysis of our suramin-selected *T*. *b*. *brucei* RNAi library revealed that RNAi fragments mapping to *Tb927*.*10*.*12940* were enriched following selection, though not to a level that fulfilled our stringency criteria [40]. Even though loss of either protein leads to a similar reduction in suramin efficacy, TbKIFC1 depletion in *12940* null 2T1 *T*. *b*. *brucei* did not enhance this effect (Fig 8C).

A preliminary western blot analysis of ^6MYC^TbKIFC1 expression following Tb927.9.8000 or Tb927.10.12940 RNAi did not show any detectable differences in expression (S6 Fig), so the basis for the putative interaction between Tb927.10.12940 and TbKIFC1 is unclear. However, the EC_50_ analyses described above suggest that the role of TbKIFC1 in apoL1 sensitivity and suramin efficacy is at least partly dependent upon Tb927.10.12940, while its impact on NHS sensitivity is independent of this putative E3-ubiquitin ligase.

## Discussion

*T*. *b*. *gambiense* and *T*. *b*. *rhodesiense* are resistant to lysis by apoL1-containing trypanolytic factors in human serum, while *T*. *b*. *brucei* is exquisitely sensitive to this attack. We report an RNAi library screen using recombinant human apoL1, which identified an extensive set of factors, including 57 novel putative apoL1 sensitivity determinants, as well as several V-ATPase subunits [36] and TbKIFC1 [37]. Prominent among the novel factors were four membrane trafficking proteins and six ubiquitin protein modifiers, including the most significant ‘hit’, a putative RING E3-ubiquitin ligase. Intriguingly, our analyses revealed that the lysosomal factors previously shown to influence the anti-trypanosomal activity of NHS have no effect on parasite sensitivity to free apoL1.

As well as the apoL1-focussed RNAi library screens described herein, several other screens have been carried out using commercially supplied [32] and freshly isolated and haptoglobin-supplemented NHS [36, 37] as selective agents. These screens led to the identification of multiple *T*. *b*. *brucei* proteins that influence trypanolysis (summarised in Table 1). Below, we discuss our results in light of the outputs from the preceding screens, and consider the implications of these data for our understanding of trypanolytic factor mode-of-action.

**Table 1:** *T*. *brucei* serum-sensitivity determinants identified by RNAi library screening.

NHS (commercial) ^a^ [32]	NHS (fresh) ^a^ [36, 37]	+haptoglobin ^a^ [36, 37]	ApoL1 (recombinant) ^a^^,^ ^b^
HpHb receptor	HpHb receptor	V-ATPase-a, -F and–H	V-ATPase-c (plus four subunits)
Inhibitor of cysteine peptidase		KIFC1	KIFC1
Lysosome-associated membrane protein, p67		V-ATPase assembly factors, PKR1 and RAV1	Putative E3-ubiquitin ligase, Tb9297.10.12940, and E2-ubiquitin conjugase, Tb927.9.8000 (plus four other putative ubiquitin modifiers)
Putative *trans*-membrane protein (Tb927.8.5240)			(Four putative membrane-trafficking proteins)

^a^ Validated hits are detailed under each screen, while key additional hits are in parentheses

^b^ See S1 Table for details

TbHpHbR-specific RNAi target fragments dominated the outputs from our original NHS RNAi library screen [32], suggesting that TLF1, which is efficiently taken up by this receptor [30], was the most abundant TLF in our NHS. To enable the identification of other *T*. *b*. *brucei* factors that promote trypanolysis, especially by TLF2 which dominates the *in vivo* human innate response to non-human infectious trypanosomes [48], Lecordier and co-workers used fresh human serum supplemented with haptoglobin to select their RNAi library [36]. However, TLF2 can also enter the parasite *via* the HpHb receptor and TLF1 can enter the parasite independently of this receptor, though in both cases entry is much less efficient [26]. Therefore, supplementation with haptoglobin will not completely ablate the effect of TLF1, though it will significantly reduce its contribution to trypanolysis. Confronted with the same challenge, we took an alternative approach; instead, selecting our *T*. *b*. *brucei* RNAi library with recombinant apoL1. As with the hyperhaptoglobinaemic serum selection strategy, selection with apoL1 identified TbKIFC1 and components of the V-ATPase complex (seven of the fourteen putative V-ATPase subunits [49] are now associated with the promotion of trypanolysis). The identification of these proteins using distinct screening approaches and the failure to identify them by RNAi library selection in NHS, highlights their importance to apoL1 and TLF2 action, and may indicate that they have a less significant role to play in TLF1-mediated trypanolysis. For example, while TbKIFC1 mediates the transit of apoL1-containing membrane to the mitochondrion [37] and its loss led to an approximately 6-fold apoL1 EC_50_ increase, there was only a marginal (approximately 1.4-fold) effect on NHS EC_50_ (see Fig 8B), explaining our inability to identify it in our TLF1-dominated NHS screen [32]. In contrast, RNAi knockdown of the V-ATPase subunit, Tb927.10.14040, revealed similar effects on sensitivity to apoL1 and our TLF1-dominated NHS, confirming that decreasing pH through the endocytic system is critically important to the trypanolytic action of TLF1, as well as apoL1 and TLF2. Therefore, while the selection of RNAi fragments targeting a specific mRNA indicates that the corresponding protein may have a role in mediating the efficacy of a selective agent, their absence is not necessarily evidence to the contrary.

**Fig 9:**
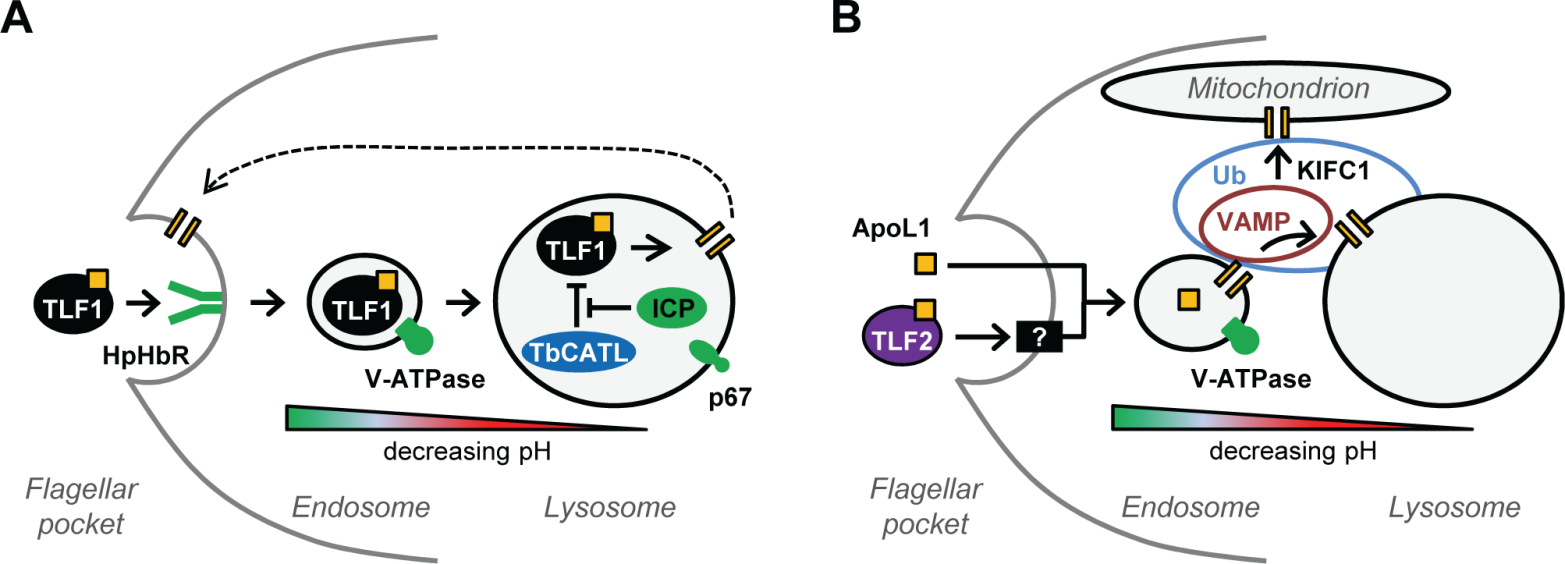
RNAi library screens reveal the distinct paths that human serum TLFs follow to instigate trypanolysis. (A) The anti-trypanosomal action of TLF1 is particularly vulnerable to changes in the surface receptor, TbHpHbR, and lysosomal function as defined by the V-ATPase, p67, ICP and TbCATL. (B) ApoL1 and TLF2 enter *T*. *brucei* via fluid phase endocytosis and an unknown mechanism (represented by the black box), respectively, and are reliant on the support of a network of sensitivity determinants, including the V-ATPase and vesicle and membrane trafficking proteins, such as TbKIFC1 and VAMP7B, which may themselves be regulated by dynamic ubiquitination. The orange bars represent membrane-integrated apoL1. The dashed line highlights the recent finding that apoL1 may also form pores in the parasite’s plasma membrane (omitted from panel (B) for clarity) [17]. Ellipses highlight possible regions of influence of the membrane trafficking (VAMP) and ubiquitination (Ub) proteins described herein.

The trypanolytic action of TLF1 is not only dependent on the parasite’s HpHb receptor [20, 30] and V-ATPase, but is also influenced by *T*. *b*. *brucei* lysosomal function (Fig 9A). Where this is impaired due to reduced levels of the lysosomal membrane protein, p67 [34], or enhanced as a consequence of ICP loss and increased TbCATL activity [24, 32], *T*. *b*. *brucei* becomes significantly less sensitive to NHS. In contrast, the data presented here demonstrate that apoL1’s ability to kill *T*. *b*. *brucei* is unaffected by the loss of p67 or ICP, or changes in TbCATL activity. Interestingly, RNAi fragments targeting p67 and ICP were not reported in the hyperhaptoglobinaemic human serum RNAi library screen described by Lecordier and co-workers [36]. Therefore, lysosomal function, as defined by these proteins, also appears to have little impact on parasite sensitivity to TLF2, as approximated for by hyperhaptoglobinaemic serum (Fig 9B).

The finding that parasite sensitivity to NHS is influenced by lysosomal factors led to the hypothesis that enhanced TbCATL activity as a consequence of ICP loss leads to destruction of TLF, apoL1 or both [32, 35], limiting the ability of apoL1 to integrate into the lysosomal membrane and form the pores that ultimately lead to trypanolysis. Alternatively, ICP loss and elevated TbCATL activity may increase the rate of TbHpHbR catabolism in the lysosome, leading to reduced TLF1 uptake. While our current data does not allow us to define the target of lysosomal TbCATL, it is clear that free apoL1 (and TLF2) is not subject to this proteolytic attack. To explain this we suggest that free apoL1 is able to integrate into the membranes of the endocytic system prior to reaching the lysosome. The lumenal pH of the eukaryotic endocytic system progressively decreases from 6–6.5 in early endosomes to 4.5–5.5 in the late endosome and lysosome [50]. Consistent with other eukaryotes, the lumenal pH of the *T*. *b*. *brucei* lysosome is approximately 4.8 [51], and it is likely that the pH of the late endosome is close to that of the lysosome. Hence, free apoL1 may be able to complete membrane integration prior to reaching the lysosome. Indeed, *in vitro* assays have shown that apoL1 is able to integrate into membranes at pH5.3 [17], higher than that recorded in the *T*. *b*. *brucei* lysosome [51]. Pre-lysosomal membrane integration by apoL1 would limit its exposure to proteolytic attack by lysosomal lumenal proteases, such as TbCATL, but endosomal-lysosomal membrane fusion would still enable it to accumulate in the lysosomal membrane (Fig 9B).

The hypothesis that apoL1 membrane integration occurs prior to the lysosome is supported by the identification of homologues of the SNARE protein, VAMP7B (Tb927.5.3560), and a putative VAMP-associated protein (Tb927.11.13230). In other eukaryotes, VAMP7B is a mediator of endosomal-lysosomal membrane fusion [52], and the VAMP-associated protein, VAP33, may have a role in vesicle trafficking through its promiscuous binding to SNARE proteins [53]. Therefore, in *T*. *b*. *brucei* these proteins may act in concert to promote the transfer of apoL1-containing membrane through the parasite’s endocytic system. Intriguingly, G1 and G2 variants of apoL1, which are associated with increased kidney disease risk in humans [54], interact with and impair VAMP8 function in human cells leading to changes in autophagosome-lysosome fusion dynamics that may underlie the observed kidney pathology [55, 56]. Furthermore, our screen identified an aminophospholipid-transporting ATPase, Tb927.11.3350, homologues of which are responsible for maintaining membrane asymmetry, which is important for efficient vesicle budding and trafficking [57].

Given the absence of free apoL1 in human serum, are these observations physiologically relevant? We do not know when, how or if apoL1 separates from TLF as it passes through the parasite’s endocytic system, though the acidic pH of the lysosome may promote TLF1-apoL1 dissociation [16]. Alternatively, the TLF1-apoL1 association may persist until TLF1 binding to the lysosomal membrane enables apoL1 integration [58]. Although TLF2 also delivers apoL1 into the parasite, its mechanism of entry and the point at which it releases its toxic payload is unknown. In the absence of a specific receptor, TLF2 may be internalised *via* the binding of its constituent IgM to the VSG coat and subsequent coat recycling [31, 36]. VSG-antibody complexes are efficiently internalised *via* hydrodynamic flow and endocytosis [59]. While the mechanism of sorting and antibody removal remain to be fully elucidated [60], this processing could conceivably lead to the release of apoL1 much earlier in the endocytic pathway, so exposing *T*. *b*. *brucei* to free apoL1 (Fig 9B).

Beyond the identity of the *T*. *b*. *brucei* proteins directly engaged in promoting the trypanolytic action of TLF and apoL1 described above, we know little about their regulation. Prominent among the apoL1 sensitivity determinants identified in our screen was a set of putative ubiquitin modifiers, including two ubiquitin ligases, a conjugase and two deubiquitinases (DUB), representing each stage of the ubiquitination and deubiquitination process. Ubiquitination is a reversible post-translational modification involved in the regulation of numerous cellular processes in eukaryotes, including protein degradation and subcellular localisation [61]. We also identified a PUB-domain containing protein (Tb927.11.6340), a candidate p97/cdc48 AAA+ ATPase cofactor [44]. Although we did not identify the *T*. *b*. *brucei* p97/cdc48 homologue (Tb927.10.5770) in our screen, this is probably unsurprising as it is expected to be involved in the regulation of many cellular processes, including membrane fusion through its interactions with various components of the ubiquitination and membrane trafficking machinery [44], so is likely highly essential. Indeed, RNAi knockdown of Tb927.10.5770 led to a severe loss of fitness in our earlier high-throughput phenotyping analysis [41].

In *T*. *b*. *brucei*, ubiquitination plays an important role in the regulation of invariant surface glycoproteins 65 and 75 [62, 63], and ISG75 contributes to the uptake of the anti-HAT drug, suramin [40], a process influenced by two putative DUBs, TbUsp7 and TbVdu1 [45]. Our data revealed an interdependence between the RING E3 ubiquitin ligase, Tb927.10.12940, and the ubiquitin E2 conjugase, Tb927.9.8000. However, we have yet to identify the target(s) of this putative ubiquitination machinery. We hypothesised that changes in ubiquitination status may be responsible for the regulation of at least some of the 63 proteins identified in our screen; a preliminary analysis revealed that 23 of these proteins, including TbKIFC1, contain high-confidence candidate ubiquitination sites. Interestingly, analysis of TbVAMP7B using the Ubpred algorithm failed to identify any high confidence ubiquitination sites, even though TbVAMP7B is influenced by TbUsp7; RNAi knockdown of this DUB leads to an approximately 40% decrease in TbVAMP7B protein levels [45]. Therefore, either the interaction between TbUsp7 and TbVAMP7B is indirect or many more of the candidate apoL1 sensitivity determinants may be subject to ubiquitination-based regulation.

Proteomic analysis of our existing RNAi (Tb927.9.8000; Tb927.10.12940) and null (Tb927.10.12940) cell lines, as well as those designed to deplete other elements of the parasite’s putative ubiquitination machinery identified in our screen, may enable the identification of their targets. However, the experimental data presented here suggests that these relationships may be complex. For example, while TbKIFC1 and Tb927.10.12940 promote apoL1 sensitivity and suramin efficacy, these two proteins have opposing effects on parasite sensitivity to NHS. Loss of TbKIFC1 reduces parasite sensitivity to NHS, but loss of either the ubiquitin ligase or the conjugase (Tb927.9.8000) sensitises *T*. *b*. *brucei* to NHS. Therefore, while ubiquitination may have a role to play in determining sensitivity to NHS, in this case, it does not promote it. In addition, loss of this putative ubiquitination machinery has no detectable effect on TbKIFC1 protein levels. However, changes in ubiquitination status may lead to subtle changes in protein levels or may affect protein function in other ways. Our EC_50_ data suggests that the influence of TbKIFC1 on apoL1 sensitivity and suramin efficacy is at least partially dependent on Tb927.10.12940, and previous work indicates that TbVAMP7B expression is influenced by ubiquitination [45] (Fig 9B).

At first glance, the identification of 63 putative apoL1 sensitivity determinants in our RNAi library screen suggests apoL1 is a trypanocide that is vulnerable to the development of resistance *via* multiple loss-of-function mutations. However, while TbKIFC1 RNAi knockdown led to an approximately six-fold increase in apoL1 EC_50_, the depletion of other factors had a more limited effect on apoL1 sensitivity (Tb927.9.8000; Tb927.10.12940), or their loss, while reducing apoL1 sensitivity, led to a significant growth defect (Tb927.9.8000; Tb927.10.14040; Tb927.10.14890). Paradoxically, the loss of some of these factors rendered *T*. *b*. *brucei* more sensitive to NHS. Taken together, these data suggest that *T*. *b*. *brucei* has little opportunity to evolve significant resistance to apoL1 *via* loss-of-function mutation. The normal association of apoL1 with two distinct carrier complexes, TLF1 and TLF2, further enhances its trypanolytic ability, providing an innate combination therapy that can exploit distinct entry mechanisms and routes through the parasite’s endocytic system to access a range of target membranes. Therefore, with the notable exception of TbHpHbR in *T*. *b*. *gambiense*, which has a reduced affinity for TLF1 [20], the development of human serum resistance in African trypanosomes is primarily dependent on gain-of-function mutation, such as the evolution of SRA in *T*. *b*. *rhodesiense* [15, 19] and TgsGP in *T*. *b*. *gambiense* [23, 24].

In summary, we have identified and validated a set of novel *T*. *b*. *brucei* apoL1 sensitivity determinants, revealing key similarities and differences with those parasite factors that support the trypanocidal effect of human serum. Further work will be required to establish the contribution of these factors to the trypanolytic action of the individual TLFs. Beyond this, our screen has highlighted an array of proteins with likely roles in the parasite’s endocytic system. At present, many of these are annotated hypothetical conserved, but future work will seek to explore their contribution to apoL1 action and to unravel their roles in the endocytic system of this divergent eukaryote.

## Materials and methods

### *T*. *b*. *brucei* strains

MITat1.2/2T1 BSF *T*. *b*. *brucei* [43] were maintained in HMI9 supplemented with 10% foetal calf serum at 37°C/5% CO_2_. Transfection was carried out in either cytomix or Tb-BSF buffer [64], for integration at the 2T1 ‘landing pad’ or native loci, respectively, using a Nucleofector (Lonza) set to programme X-001. Transformants were selected in 2.5 μg.ml^-1^ hygromycin, 1 μg.ml^-1^ G418 or 10 μg.ml^-1^ blasticidin, as appropriate. The BSF *T*. *b*. *brucei* RNAi library was maintained in 1 μg.ml^-1^ phleomycin and 5 μg.ml^-1^ blasticidin. For growth assays, cells were seeded at ~10^5^ ml^-1^, counted using a haemocytometer, and diluted back every 24 hours, as necessary, for up to six days in the absence of antibiotics.

### ApoL1 and NHS sensitivity assays

ApoL1 was prepared as previously described [38]. NHS (pooled mixed gender; 0.2 μm filtered; stored at -80°C in 1 ml aliquots) was purchased from Sera Laboratories International; this batch was used in our previous NHS RNAi library screen [32]. To determine the half maximal effective concentration (EC_50_) of NHS and recombinant apoL1, BSF *T*. *b*. *brucei* were seeded at 2x10^3^ ml^-1^ in 96-well plates in a 2-fold dilution series of NHS or recombinant apoL1, starting from 0.01% or 10 μg.ml^-1^, respectively; assays were carried out in the absence of antibiotics. After ~3 days growth, resazurin (Sigma) in PBS was added to a final concentration of 12.5 μg.ml^-1^ per well, and the plates incubated for a further 6 hours at 37°C. Fluorescence was determined using a fluorescence plate reader (Molecular Devices) at an excitation wavelength of 530 nm, an emission wavelength of 585 nm and a filter cut-off of 570 nm [65]. Data were processed in Excel, and non-linear regression analysis carried out in GraphPad Prism. The short-term kinetics of killing in high concentration apoL1 (10 μg.ml^-1^) was determined as previously described [37].

### BSF *T*. *b*. *brucei* RNAi library screening and RIT-seq

RNA library screening was carried out as previously described [39]. Briefly, library expression was induced in 1 μg.ml^-1^ tetracycline for 24 hours prior to selection in 2X EC_50_ recombinant apoL1. Cell density was counted daily using a haemocytometer and the total cell number diluted to no less than 20 million in 100 ml media; induction and apoL1 selection were maintained throughout. Once robust growth had been achieved for at least two days, genomic DNA was prepared for RNAi target identification. The RNAi cassettes remaining in the apoL1-selected RNAi libraries were specifically amplified from genomic DNA using the LIB2F/LIB2R primers, and sequenced on an Illumina MiSeq platform.

To prepare sequencing libraries, 300 ng of each RNAi library cassette PCR was fragmented with a Covaris Ultrasonicator using settings optimised to produce a fragment size of <500 bp (peak incident factor = 50, duty factor = 20%, cycles per burst = 200, for 3 minutes). Fragment size was confirmed using a Bioanalyzer and High Sensitivity DNA kit (Agilent). Sequencing libraries were prepared using the TruSeq Nano DNA library preparation kit (Illumina) according to manufacturer’s instructions, including a final PCR step to enrich for DNA fragments with the sequencing adaptor ligated onto each end of the fragment. The final size range of each sequencing library was measured on a Bioanalyzer, and quantified using a fluorometric quantification assay (Qubit) and quantitative PCR (KAPA Library Quantification kit). Library DNA concentration was normalised to 10 nM and individual libraries pooled. The pooled libraries were denatured and diluted to 5 pM, according to manufacturer’s recommendations (Ilumina MiSeq Reagents kit v2 500 cycles). Pooled libraries were spiked with 50% PhiX (Illumina) due to the low complexity of the samples and a total of 10 pM DNA was loaded onto a MiSeq flow cell for paired-end sequencing.

The sequenced RNAi target fragments were mapped against the *T*. *b*. *brucei* strain TREU927 reference genome (release 8.0) using the ‘alternative protocol’, as described [39]. Briefly, mapping was carried out using Bowtie2 [66] set to ‘very sensitive local’ alignment and output SAM files were processed using SAMtools [67]. The resultant BAM files were viewed against the reference genome in the Artemis genome browser [68]. Reads containing the RNAi construct-specific 14-base barcode were identified using a custom script [39], and this subset of reads were mapped, as above. Plots were derived using the Artemis graph tool and processed in Adobe Photoshop Elements 8.0. Stacks of reads that included the 14-base barcode on the positive strand were used to define RNAi target fragment junctions and to assign high-confidence hits as those identified by >1 RNAi target fragment.

### Plasmid and strain construction

Stemloop RNAi constructs targeting Tb927.10.14040, Tb927.10.14890, Tb927.9.8000 and Tb927.10.12940 were assembled in pRPa^iSL^ [42]. RNAi targeting fragments were designed using the RNAit primer design algorithm to minimise off-target effects [69]. pRPa^iSL^ constructs were linearised with *Asc*I prior to transfection and targeted integration at the rDNA spacer ‘landing pad’ locus in 2T1 BSF *T*. *b*. *brucei* [43]. Constructs to enable *C*- or *N*-terminal protein tagging of Tb927.10.14040 (x^12MYC^; *Nsi*I) and Tb927.9.8000 (^GFP^x; *Acc*65I) at the endogenous loci were assembled in pNAT^BSD^ [42]. TbKIFC1 (Tb927.10.14890) was *N*-terminally tagged at the endogenous locus using pNAT^BSD 6MYC^TbKIFC1 (*Sph*I); plasmid provided by Klaus Ersfeld, University of Bayreuth, Germany. All constructs were linearised by digestion with the respective restriction endonuclease prior to transfection into 2T1 BSF *T*. *b*. *brucei*.

For deletion of both copies of *Tb927*.*10*.*12940*, targeting fragments were cloned into pBSD, and the blasticidin-S-deaminase cassette was then replaced with a neomycin phosphotransferase cassette to generate pBSDΔ12940 and pNPTΔ12940 constructs. A full-length copy of *Tb927*.*10*.*12940* was cloned into pRPa via *Hin*dIII/*Bgl*II (full length; untagged) or *Hin*dIII/*Xba*I (minus stop codon; *C*-terminal GFP) to enable protein overexpression in wild type and *Tb927*.*10*.*12940* null 2T1 BSF *T*. *b*. *brucei*. Details of all primers are available on request. Expression of tagged protein was analysed by SDS-PAGE and western blotting with rabbit anti-GFP (Molecular Probes) and mouse anti-cMyc (Source BioScience), using standard protocols [70]. The subcellular localisation of Tb927.10.12940^GFP^ and p67 was assessed following cell fixation in 2% paraformaldehyde in PBS, permeabilisation in 0.5% triton-X100 and incubation with anti-GFP and anti-p67 (Jay Bangs, SUNY Buffalo); bound primary antibodies were detected with FITC-anti-rabbit and rhodamine-anti-mouse antibodies (Pierce). Slides were mounted in vectorshield (Vector Laboratories) containing 4',6-diamidino-2-phenylindole (DAPI) to enable visualisation of nuclear and kinetoplast DNA.

### cDNA synthesis and qPCR analysis

As Tb927.10.12940 tagged at the native locus was undetectable by western blot, we confirmed knockdown by RT-qPCR. For each cell line and treatment (uninduced and induced), 2 μg RNA was DNase-treated and reverse-transcribed using the Superscript VILO cDNA synthesis kit (Invitrogen). 100 ng (RNA-equivalent) cDNA was subjected to qPCR using the Quantitect SYBR Green PCR kit (Qiagen) and primer pairs specific for telomerase reverse transcriptase (TERT; Tb927.11.10190) and Tb927.10.12940 (details of primer sequences are available on request). TERT was used as a reference for normalisation of gene expression, as previously described [71]. qPCR reactions were carried out in a Rotor-gene 3000 (Corbett Research), using the following cycling conditions: 95°C (15 minutes), followed by 40 cycles of 94°C (15 seconds), 58°C (30 seconds), and 72°C (30 seconds). Standard curves, derived from a series of 10-fold dilutions of the target PCR products, were used to determine reaction efficiency. Fold-change in gene expression was calculated by the ΔΔCt method [72].

## Supporting information

S1 Fig The putative V_0_c V-ATPase subunit (Tb927.10.14040) promotes apoL1 and NHS-mediated killing of *T*. *b*. *brucei*. (A) Tb927.10.14040 RNAi knockdown in *T*. *b*. *brucei* leads to a significant growth defect; data derived from three independent cell lines. Inset shows depletion of Tb927.10.14040^12MYC^ following RNAi induction in tetracycline (tet) for 24 hours; Coomassie-stained gel shown for loading. (B) ApoL1 and (C) NHS sensitivity following Tb927.10.14040 RNAi depletion. Three independent Tb927.10.14040 RNAi cell lines were induced for 24 hours in 2 ng.ml^-1^ tetracycline before exposure to 10 ug.ml^-1^ apoL1 or 0.1% NHS for 24 hours under the same inducing conditions; cell densities were counted at the indicated times using a haemocytometer. Population growth is presented relative to the corresponding untreated culture; error bars, standard deviation; *P*-values derived from paired students t-test.
(TIF)

S2 Fig p67 and ICP, fail to fulfil the stringency criteria for inclusion in the apoL1 RNAi library ‘hit’ list. Histograms showing RNAi target fragment mapping to (A) the chromosome-5 region flanking *Tb927*.*5*.*1810* and *Tb927*.*5*.*1830* (lysosomal-associated membrane protein, *p67*) and (B) the chromosome-8 region flanking *Tb927*.*8*.*6450* (‘inhibitor of cysteine peptidase’, *ICP*) following RNAi library selection in apoL1 and normal human serum (NHS); RNAi construct-specific barcode-containing reads presented as RPKM (plus 0.1) as per Fig 3A.
(TIF)

S3 Fig Tb927.10.12940 depletion and Tb927.10.12940^GFP^ subcellular localisation. (A) Cumulative growth following RNAi knockdown of Tb927.10.12940. Inset shows RT-qPCR quantification of Tb927.10.12940 depletion following targeted RNAi knockdown; three independent cell lines induced in 1 μg.ml^-1^ tetracycline; red dashed line corresponds to RNA levels in the absence of RNAi induction. (B) Immunofluorescence localisation of Tb927.10.12940^GFP^; counter-staining with the DNA intercalating dye, DAPI, reveals the kinetoplast (k) and nucleus (n).
(TIF)

S4 Fig Tb927.9.8000 depletion and *Tb927*.*10*.*12940* deletion does not have an additive effect on *T*. *b*. *brucei* population growth. (A) Specific RNAi depletion of ^GFP^8000 by western blotting; Coomassie-stained gel shown for loading. (B) Cumulative growth following RNAi knockdown of Tb927.9.8000 in wild type 2T1 *T*. *b*. *brucei*; three independent cell lines induced in 1 μg.ml^-1^ tetracycline. (C) Cumulative growth following RNAi knockdown of Tb927.9.8000 in *12940* null 2T1 *T*. *b*. *brucei*; four independent cell lines induced in 1 μg.ml^-1^ tetracycline. (D) Chart summarising the impact on population doubling times of Tb927.9.8000 RNAi knockdown in wild type and *Tb927*.*10*.*12940* null 2T1 *T*. *b*. *brucei*; data derived from (B) and (C). Error bars, standard deviation; *P*-values derived from paired students t-test.
(TIF)

S5 Fig TbKIFC1 depletion and *Tb927*.*10*.*12940* deletion does not have an additive effect on *T*. *b*. *brucei* population growth. (A) Specific RNAi depletion of ^6MYC^TbKIFC1; Coomassie-stained gel shown for loading. (B) Cumulative growth following RNAi knockdown of TbKIFC1 in 2T1 *T*. *b*. *brucei*; three independent cell lines induced in 1 μg.ml^-1^ tetracycline. (C) Cumulative growth following RNAi knockdown of TbKIFC1 in *12940* null 2T1 *T*. *b*. *brucei*; four independent cell lines induced in 1 μg.ml^-1^ tetracycline. (D) Chart summarising the impact on population doubling times of TbKIFC1 RNAi knockdown in wild type and *Tb927*.*10*.*12940* null 2T1 *T*. *b*. *brucei*; data derived from (B) and (C). Error bars, standard deviation; *P*-values derived from paired students t-test.
(TIF)

S6 Fig Depletion of Tb927.9.8000 or Tb927.10.12940 has no significant effect on ^6MYC^TbKIFC1 expression. ^6MYC^TbKIFC1 expression following (A) Tb927.10.12940 and (B) Tb927.9.8000 RNAi knockdown; Coomassie-stained gels shown for loading.
(TIF)

S1 Table Genes represented by >99 RNAi construct-specific barcode-containing reads per kilobase per transcript following RNAi library selection in 2X EC_50_ human apoL1. Coloured as per Fig 2. ^a^ Annotations derived from GeneDB, http://www.genedb.org/Homepage/Tbruceibrucei927, 16^th^ November 2016. ^b^ ‘Tagged reads’ incorporate a 14-base RNAi construct barcode, GTGAGGCCTCGCGA. ^c^ ‘Tagged reads/kb/mRNA’ plotted in Fig 2A. ^d^ BSF *T*. *b*. *brucei* RNAi library fitness ratio derived from reference [41]; three-day induced read count divided by uninduced read count; the lower the number the greater the fitness cost following RNAi depletion, whereas a number >1 indicates a gain of fitness.
(XLSX)

S2 Table RPKM values for genes detailed in S1 Table following RNAi library selection with 2X EC_50_ human apoL1 or normal human serum (NHS). Coloured as per Fig 3A. ^a^ Annotations derived from GeneDB, http://www.genedb.org/Homepage/Tbruceibrucei927, 16^th^ November 2016. ^b^ ‘Tagged reads’ incorporate a 14-base RNAi construct barcode, GTGAGGCCTCGCGA. ^c^ Sequencing data from reference [32] reanalysed to quantify the number of tagged reads per kilobase per predicted transcript; output converted to RPKM.
(XLSX)

S3 Table RPKM values for the top ‘hits’ identified following RNAi library selection with 2X EC_50_ normal human serum (NHS) and the corresponding RPKM values following selection in human apoL1. Coloured as per Fig 3A, highlighting the previously validated *T*. *b*. *brucei* determinants of human serum sensitivity described in reference [32]. ^a^ Annotations derived from GeneDB, http://www.genedb.org/Homepage/Tbruceibrucei927, 16^th^ November 2016. ^b^ ‘Tagged reads’ incorporate a 14-base RNAi construct barcode, GTGAGGCCTCGCGA. ^c^ Sequencing data from reference [32] reanalysed to quantify the number of tagged reads per kilobase per predicted transcript; output converted to RPKM. ^d^ Number of independent RNAi fragments targeting each transcript following BSF *T*. *b*. *brucei* RNAi library selection in NHS; see reference [32].
(XLSX)

S4 Table UbPred analysis identifies putative ubiquitination sites in putative apoL1-sensitivity determinants. ^a^ Annotations derived from GeneDB, http://www.genedb.org/Homepage/Tbruceibrucei927, 16^th^ November 2016. ^b^ Confidence assigned depending on the UbPred score for each lysine in a protein sequence; low, 0.62 ≤ s ≤ 0.69; medium, 0.69 ≤ s ≤ 0.84; high, 0.84 ≤ s ≤ 1.00 [46].
(XLSX)
