# Supplementary figures and images for: Decoding the network of Trypanosoma brucei proteins that determines sensitivity to apolipoprotein-L1
Source: PLoS Pathog. 2018 Jan 18;14(1):e1006855. doi: 10.1371/journal.ppat.1006855 (PMC5790291; doi:10.1371/journal.ppat.1006855)

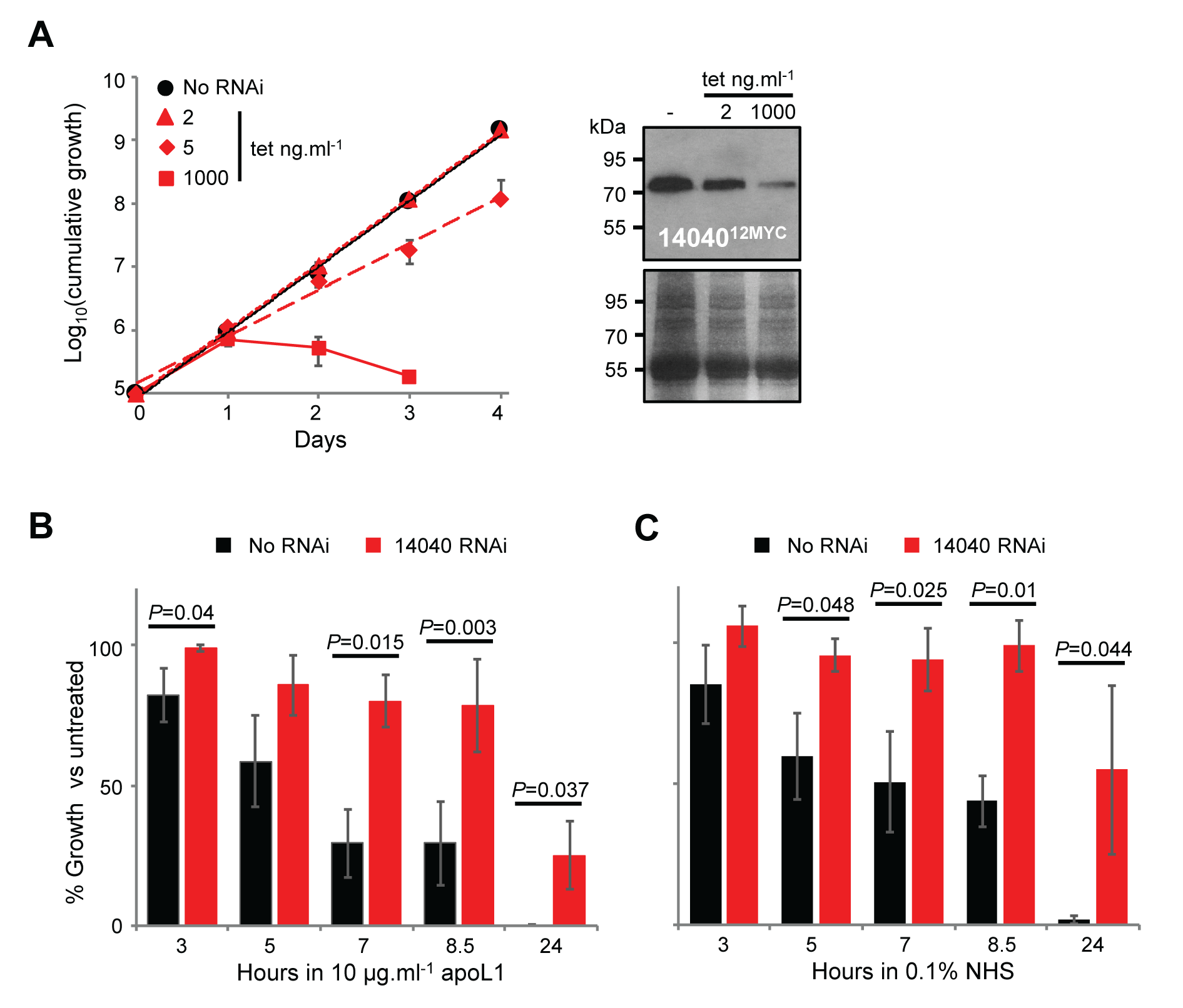

Supplement: S1 Fig — (A) Tb927.10.14040 RNAi knockdown in T. b. brucei leads to a significant growth defect; data derived from three independent cell lines. Inset shows depletion of Tb927.10.1404012MYC following RNAi induction in tetracycline (tet) for 24 hours; Coomassie-stained gel shown for loading. (B) ApoL1 and (C) NHS sensitivity following Tb927.10.14040 RNAi depletion. Three independent Tb927.10.14040 RNAi cell lines were induced for 24 hours in 2 ng.ml-1 tetracycline before exposure to 10 ug.ml-1 apoL1 or 0.1% NHS for 24 hours under the same inducing conditions; cell densities were counted at the indicated times using a haemocytometer. Population growth is presented relative to the corresponding untreated culture; error bars, standard deviation; P-values derived from paired students t-test. (TIF) [file ppat.1006855.s001.tif]

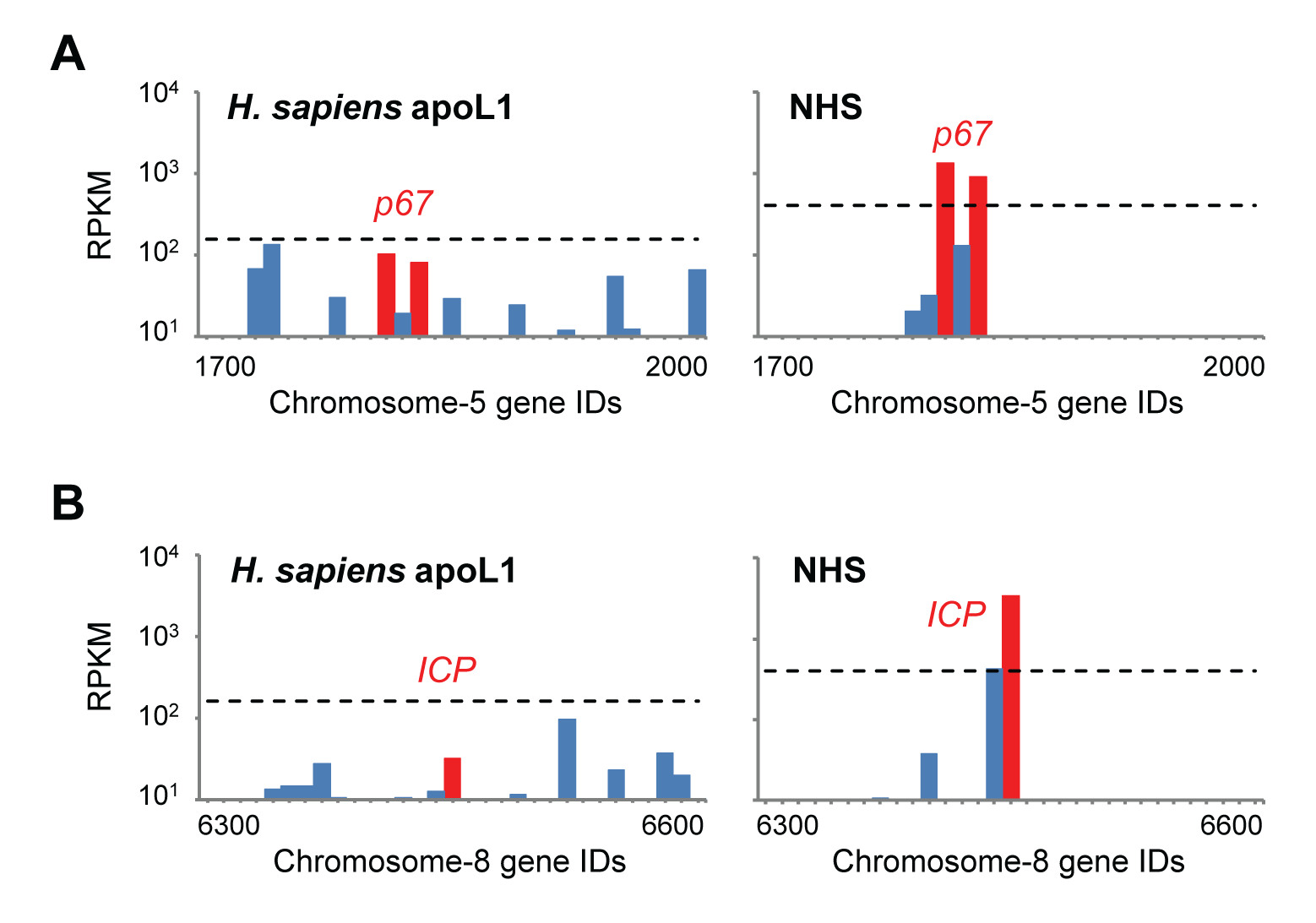

Supplement: S2 Fig — Histograms showing RNAi target fragment mapping to (A) the chromosome-5 region flanking Tb927.5.1810 and Tb927.5.1830 (lysosomal-associated membrane protein, p67) and (B) the chromosome-8 region flanking Tb927.8.6450 (‘inhibitor of cysteine peptidase’, ICP) following RNAi library selection in apoL1 and normal human serum (NHS); RNAi construct-specific barcode-containing reads presented as RPKM (plus 0.1) as per Fig 3A. (TIF) [file ppat.1006855.s002.tif]

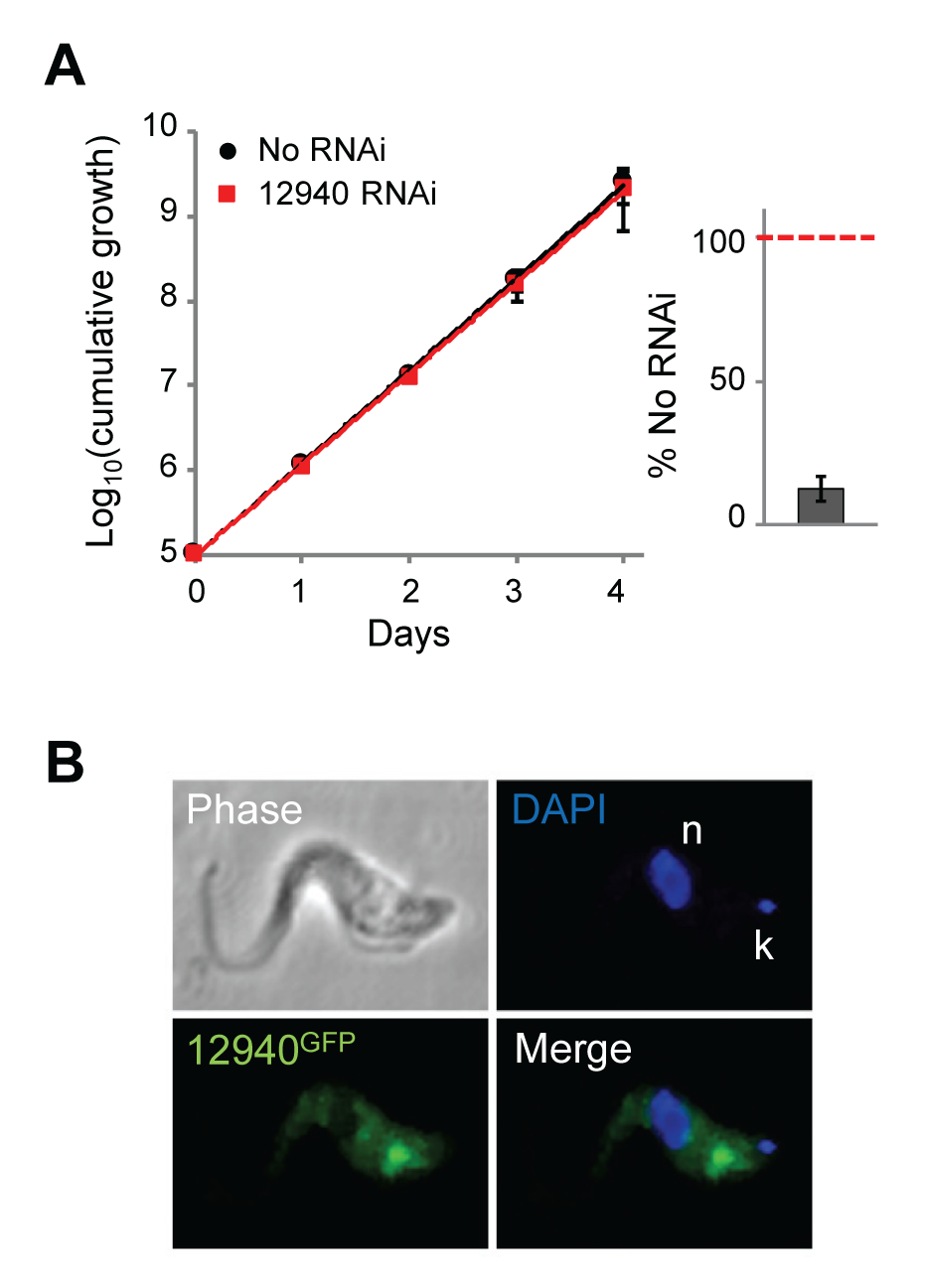

Supplement: S3 Fig — (A) Cumulative growth following RNAi knockdown of Tb927.10.12940. Inset shows RT-qPCR quantification of Tb927.10.12940 depletion following targeted RNAi knockdown; three independent cell lines induced in 1 μg.ml-1 tetracycline; red dashed line corresponds to RNA levels in the absence of RNAi induction. (B) Immunofluorescence localisation of Tb927.10.12940GFP; counter-staining with the DNA intercalating dye, DAPI, reveals the kinetoplast (k) and nucleus (n). (TIF) [file ppat.1006855.s003.tif]

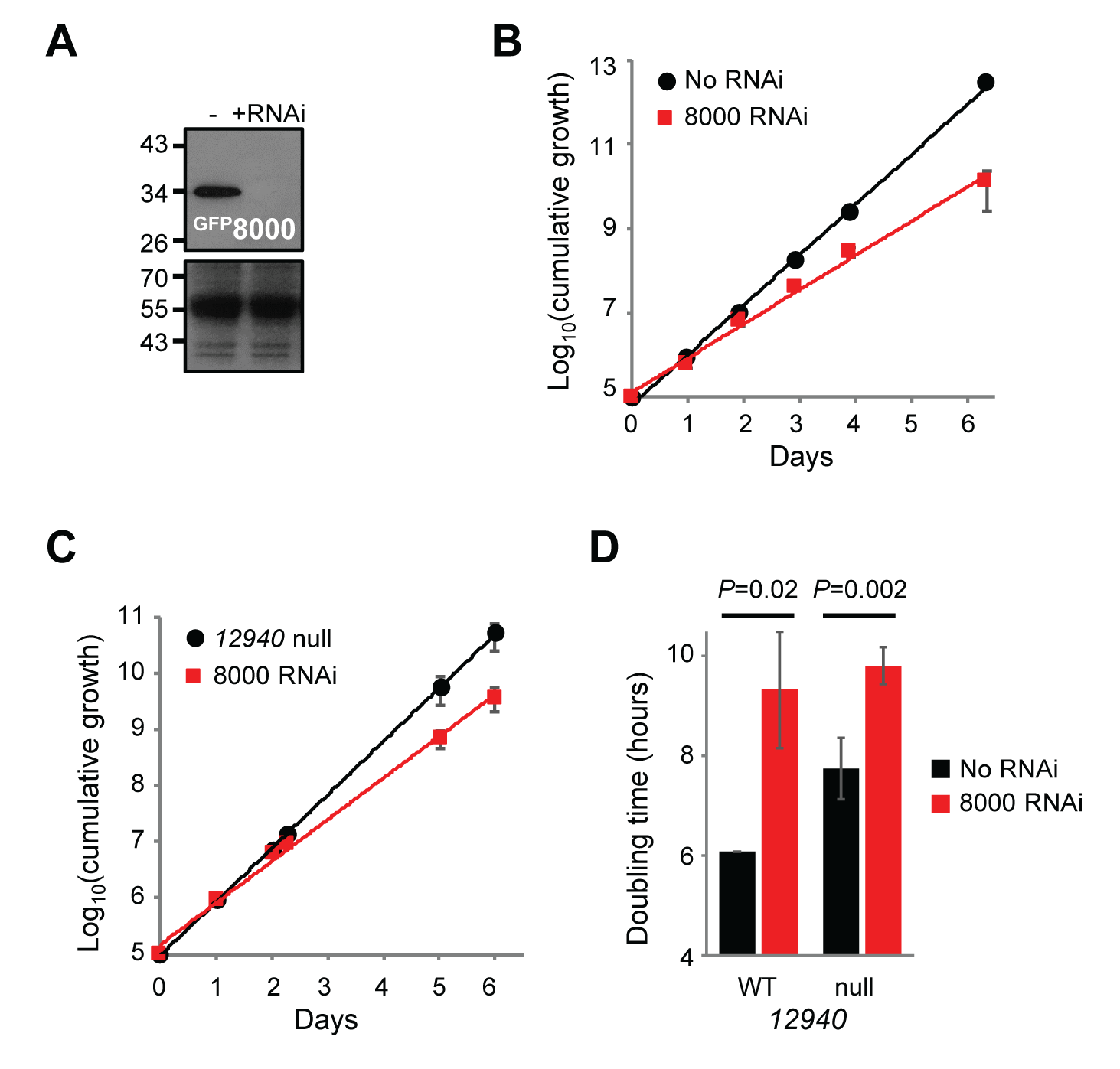

Supplement: S4 Fig — (A) Specific RNAi depletion of GFP8000 by western blotting; Coomassie-stained gel shown for loading. (B) Cumulative growth following RNAi knockdown of Tb927.9.8000 in wild type 2T1 T. b. brucei; three independent cell lines induced in 1 μg.ml-1 tetracycline. (C) Cumulative growth following RNAi knockdown of Tb927.9.8000 in 12940 null 2T1 T. b. brucei; four independent cell lines induced in 1 μg.ml-1 tetracycline. (D) Chart summarising the impact on population doubling times of Tb927.9.8000 RNAi knockdown in wild type and Tb927.10.12940 null 2T1 T. b. brucei; data derived from (B) and (C). Error bars, standard deviation; P-values derived from paired students t-test. (TIF) [file ppat.1006855.s004.tif]

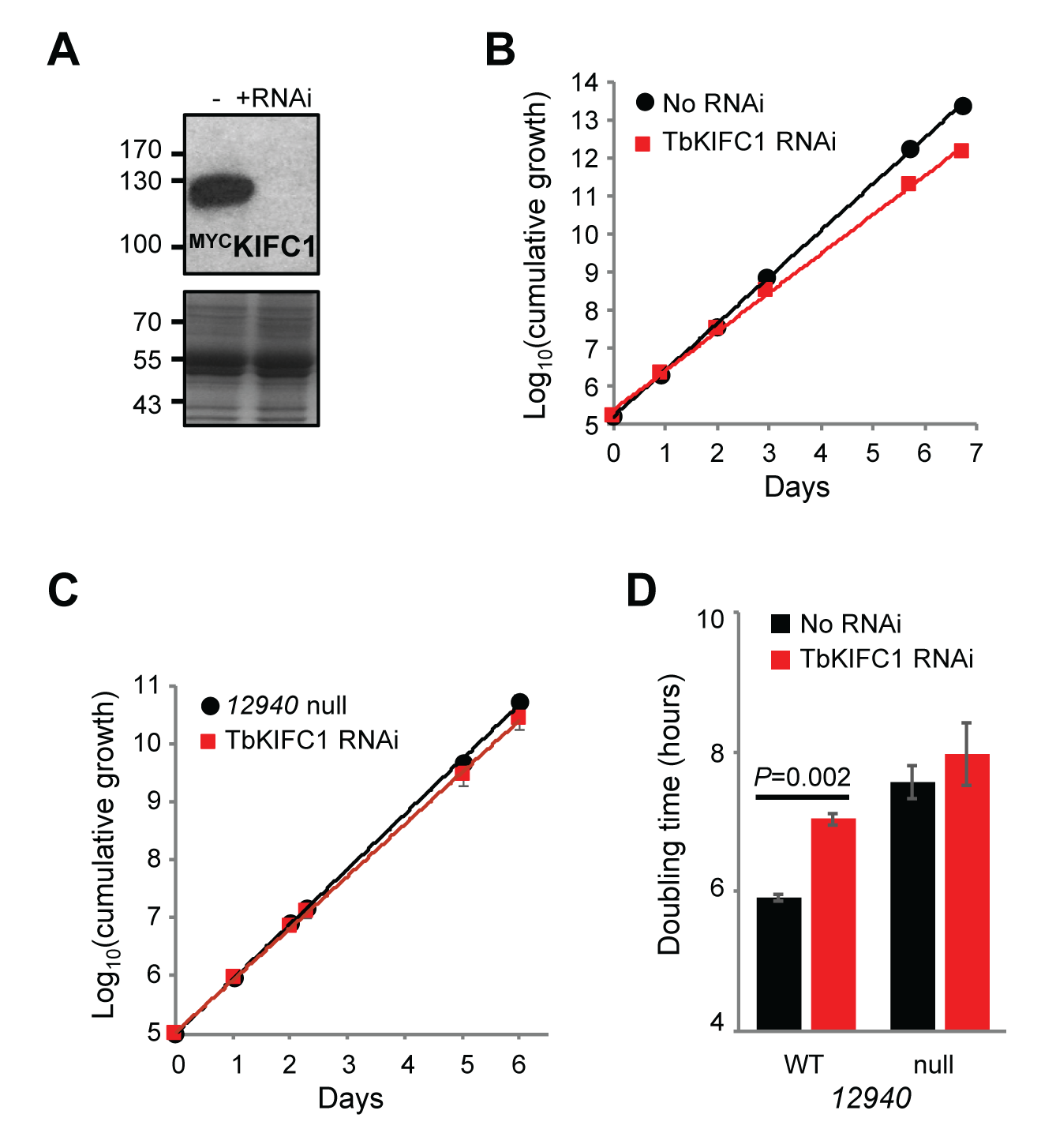

Supplement: S5 Fig — (A) Specific RNAi depletion of 6MYCTbKIFC1; Coomassie-stained gel shown for loading. (B) Cumulative growth following RNAi knockdown of TbKIFC1 in 2T1 T. b. brucei; three independent cell lines induced in 1 μg.ml-1 tetracycline. (C) Cumulative growth following RNAi knockdown of TbKIFC1 in 12940 null 2T1 T. b. brucei; four independent cell lines induced in 1 μg.ml-1 tetracycline. (D) Chart summarising the impact on population doubling times of TbKIFC1 RNAi knockdown in wild type and Tb927.10.12940 null 2T1 T. b. brucei; data derived from (B) and (C). Error bars, standard deviation; P-values derived from paired students t-test. (TIF) [file ppat.1006855.s005.tif]

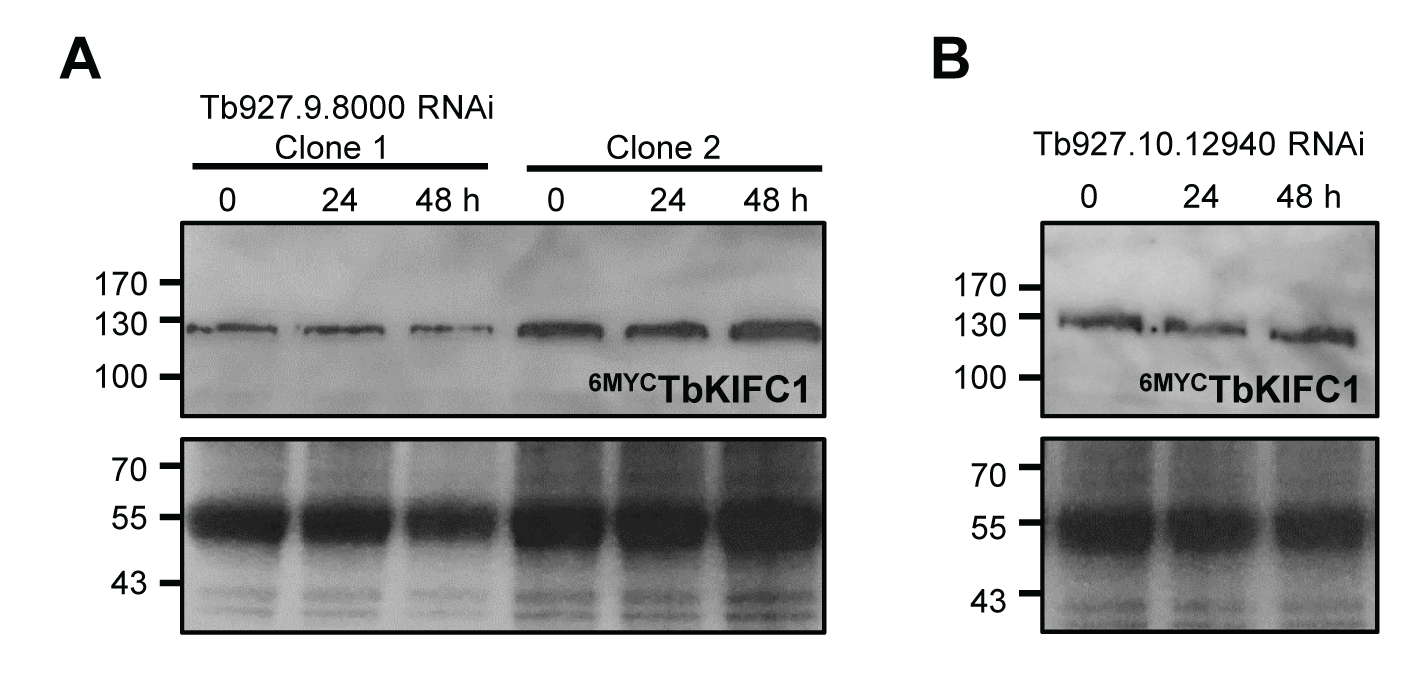

Supplement: S6 Fig — 6MYCTbKIFC1 expression following (A) Tb927.10.12940 and (B) Tb927.9.8000 RNAi knockdown; Coomassie-stained gels shown for loading. (TIF) [file ppat.1006855.s006.tif]
